# A user-friendly tool for cloud-based whole slide image segmentation with examples from renal histopathology

**DOI:** 10.1038/s43856-022-00138-z

**Published:** 2022-08-19

**Authors:** Brendon Lutnick, David Manthey, Jan U. Becker, Brandon Ginley, Katharina Moos, Jonathan E. Zuckerman, Luis Rodrigues, Alexander J. Gallan, Laura Barisoni, Charles E. Alpers, Xiaoxin X. Wang, Komuraiah Myakala, Bryce A. Jones, Moshe Levi, Jeffrey B. Kopp, Teruhiko Yoshida, Jarcy Zee, Seung Seok Han, Sanjay Jain, Avi Z. Rosenberg, Kuang Yu. Jen, Pinaki Sarder, the Kidney Precision Medicine Project, Brendon Lutnick, Brandon Ginley, Richard Knight, Stewart H. Lecker, Isaac Stillman, Steve Bogen, Afolarin A. Amodu, Titlayo Ilori, Insa Schmidt, Shana Maikhor, Laurence H. Beck, Ashish Verma, Joel M. Henderson, Ingrid Onul, Sushrut Waikar, Gearoid M. McMahon, Astrid Weins, Mia R. Colona, M. Todd Valerius, Nir Hacohen, Paul J. Hoover, Anna Greka, Jamie L. Marshall, Mark Aulisio, Yijiang M. Chen, Andrew Janowczyk, Catherine Jayapandian, Vidya S. Viswanathan, William S. Bush, Dana C. Crawford, Anant Madabhushi, John O’toole, Emilio Poggio, John Sedor, Leslie Cooperman, Stacey Jolly, Leal Herlitz, Jane Nguyen, Agustin Gonzalez-Vicente, Ellen Palmer, Dianna Sendrey, Jonathan Taliercio, Lakeshia Bush, Kassandra Spates-Harden, Carissa Vinovskis, Petter M. Bjornstad, Laura Pyle, Paul Appelbaum, Jonathan M. Barasch, Andrew S. Bomback, Vivette D. D’Agati, Krzysztof Kiryluk, Karla Mehl, Pietro A. Canetta, Ning Shang, Olivia Balderes, Satoru Kudose, Theodore Alexandrov, Helmut Rennke, Tarek M. El-Achkar, Yinghua Cheng, Pierre C. Dagher, Michael T. Eadon, Kenneth W. Dunn, Katherine J. Kelly, Timothy A. Sutton, Daria Barwinska, Michael J. Ferkowicz, Seth Winfree, Sharon Bledsoe, Marcelino Rivera, James C. Williams, Ricardo Melo Ferreira, Katy Borner, Andreas Bueckle, Bruce W. Herr, Ellen M. Quardokus, Elizabeth Record, Jing Su, Debora Gisch, Stephanie Wofford, Yashvardhan Jain, Chirag R. Parikh, Celia P. Corona-Villalobos, Steven Menez, Yumeng Wen, Camille Johansen, Sylvia E. Rosas, Neil Roy, Mark Williams, Jennifer Sun, Joseph Ardayfio, Jack Bebiak, Keith Brown, Catherine E. Campbell, John Saul, Anna Shpigel, Christy Stutzke, Robert Koewler, Taneisha Campbell, Lynda Hayashi, Nichole Jefferson, Glenda V. Roberts, Roy Pinkeney, Evren U. Azeloglu, Cijang He, Ravi Iyengar, Jens Hansen, Yuguang Xiong, Pottumarthi Prasad, Anand Srivastava, Brad Rovin, Samir Parikh, John P. Shapiro, Sethu M. Madhavan, Christopher R. Anderton, Ljiljana Pasa-Tolic, Dusan Velickovic, Jessica Lukowski, George Holt Oliver, Olga Troyanskaya, Rachel Sealfon, Aaron Wong, Katherine R. Tuttle, Ari Pollack, Yury Goltsev, Kun Zhang, Blue B. Lake, Zoltan G. Laszik, Garry Nolan, Patrick Boada, Minnie Sarwal, Kavya Anjani, Tara Sigdel, Tariq Mukatash, Paul J. Lee, Rita R. Alloway, E. Steve Woodle, Ashley R. Burg, Adele Rike, Tiffany Shi, Heather Ascani, Ulysses G. J. Balis, Jeffrey B. Hodgin, Matthias Kretzler, Chrysta Lienczewski, Laura H. Mariani, Rajasree Menon, Becky Steck, Yougqun He, Edgar Otto, Jennifer Schaub, Victoria M. Blanc, Sean Eddy, Ninive C. Conser, Jinghui Luo, Renee Frey, Paul M. Palevsky, Matthew Rosengart, John A. Kellum, Daniel E. Hall, Parmjeet Randhawa, Mitchell Tublin, Raghavan Murugan, Michele M. Elder, James Winters, Tina Vita, Filitsa Bender, Roderick Tan, Matthew Gilliam, Kristina N. Blank, Jonas Carson, Ian H. De Boer, Ashveena L. Dighe, Jonathan Himmelfarb, Sean D. Mooney, Stuart Shankland, Kayleen Williams, Christopher Park, Frederick Dowd, Robyn L. McClelland, Stephen Daniel, Andrew N. Hoofnagle, Adam Wilcox, Stephanie M. Grewenow, Ashley Berglund, Christine Limonte, Kasra Rezaei, Ruikang Wang, Jamie Snyder, Brooke Berry, Yunbi Nam, Natalya Sarkisova, Shweta Bansal, Kumar Sharma, Manjeri Venkatachalam, Guanshi Zhang, Annapurna Pamreddy, Hongping Ye, Richard Montellano, Robert D. Toto, Miguel Vazquez, Simon C. Lee, R. Tyler Miller, Orson W. Moe, Jose Torrealba, Nancy Wang, Asra Kermani, Kamalanathan Sambandam, Harold Park, S. Susan Hedayati, Christopher Y. Lu, Natasha Wen, Jiten Patel, Anil Pillai, Dianbo Zhang, Mujeeb Basit, Allen H. Hendricks, Richard M. Caprioli, Nathan Patterson, Kavya Sharman, Jeffrey M. Spraggins, Raf Van de Plas, Anitha Vijayan, Joseph P. Gaut, Jeanine Basta, Sabine M. Diettman, Michael I. Rauchman, Dennis Moledina, Francis P. Wilson, Ugochukwu Ugwuowo, Tanima Arora, Melissa M. Shaw, Lloyd G. Cantley, Vijaykumar R. Kakade, Angela Victoria-Castro

**Affiliations:** 1 Department of Pathology and Anatomical Sciences, SUNY Buffalo, Buffalo, USA; 2 Kitware Incorporated, Clifton Park, USA; 3 Institute of Pathology, University Hospital Cologne, Cologne, Germany; 4 Department of Pathology and Laboratory Medicine, University of California at Los Angeles, Los Angeles, USA; 5 University Clinic of Nephrology, Faculty of Medicine, University of Coimbra, Coimbra, Portugal; 6 Department of Pathology, Medical College of Wisconsin, Milwaukee, USA; 7 Departments of Pathology and Medicine, Duke University, Durham, USA; 8 Department of Laboratory Medicine and Pathology, University of Washington, Seattle, USA; 9 Departments of Biochemistry and Molecular & Cellular Biology, Georgetown University, Washington, DC USA; 10 Department of Pharmacology and Physiology, Georgetown University, Washington, DC USA; 11 Kidney Disease Section, NIDDK, NIH, Bethesda, USA; 12 Department of Biostatistics, Epidemiology, & Informatics, University of Pennsylvania, Philadelphia, USA; 13 Department of Internal Medicine, Seoul National University College of Medicine, Seoul, South Korea; 14 Department of Medicine, Nephrology, Washington University School of Medicine, St. Louis, USA; 15 Department of Pathology, Johns Hopkins University, Baltimore, USA; 16 Department of Pathology and Laboratory Medicine, University of California at Davis, Sacramento, USA; 17 SUNY Buffalo, Buffalo, USA; 18 American Association for Kidney Patients, Tampa, USA; 19 Beth Israel Deaconess, Boston, USA; 20 Boston Cell Standards, Boston, USA; 21 Boston Medical Center, Boston, USA; 22 Boston University, Boston, USA; 23 Brigham & Women’s Hospital, Boston, USA; 24 Broad Institute, Boston, USA; 25 Case Western Reserve University, Cleveland, USA; 26 Cleveland Clinic, Cleveland, USA; 27 Colorado Children’s Hospital, Aurora, USA; 28 Columbia University, New York, USA; 29 European Molecular Biology Laboratory, Heidelberg, Germany; 30 Harvard University, Boston, USA; 31 Indiana University, Bloomington, USA; 32 Johns Hopkins University, Baltimore, USA; 33 Joslin Diabetes Center, Boston, USA; 34 KPMP Patient Partner, Seattle, USA; 35 Mount Sinai, New York, USA; 36 Northshore University, Evanston, USA; 37 Northwestern University, Chicago, USA; 38 Ohio State University, Columbus, USA; 39 Pacific Northwest National Laboratories, Seattle, USA; 40 Parkland Hospital, Dallas, USA; 41 Princeton University, New Jersey, USA; 42 Providence Health, Portland, USA; 43 Seattle Children’s Hospital, Seattle, USA; 44 Stanford University, Stanford, USA; 45 University of California, San Diego, USA; 46 University of California, San Francisco, USA; 47 University of Cincinnati, Cincinnati, USA; 48 University of Michigan, Michigan, USA; 49 University of Pittsburgh, Pittsburgh, USA; 50 University of Washington, Seattle, USA; 51 University of Texas Health San Antonio, San Antonio, USA; 52 University of Texas Southwestern, Dallas, USA; 53 Vanderbilt University, Nashville, USA; 54 Washington University School of Medicine, St. Louis, USA; 55 Yale University, New Haven, USA

**Keywords:** End-stage renal disease, Computational biology and bioinformatics

## Abstract

**Background:**

Image-based machine learning tools hold great promise for clinical applications in pathology research. However, the ideal end-users of these computational tools (e.g., pathologists and biological scientists) often lack the programming experience required for the setup and use of these tools which often rely on the use of command line interfaces.

**Methods:**

We have developed *Histo-Cloud*, a tool for segmentation of whole slide images (WSIs) that has an easy-to-use graphical user interface. This tool runs a state-of-the-art convolutional neural network (CNN) for segmentation of WSIs in the cloud and allows the extraction of features from segmented regions for further analysis.

**Results:**

By segmenting glomeruli, interstitial fibrosis and tubular atrophy, and vascular structures from renal and non-renal WSIs, we demonstrate the scalability, best practices for transfer learning, and effects of dataset variability. Finally, we demonstrate an application for animal model research, analyzing glomerular features in three murine models.

**Conclusions:**

*Histo-Cloud* is open source, accessible over the internet, and adaptable for segmentation of any histological structure regardless of stain.

## Plain language summary

Artificial intelligence (AI) is the ability of a computer to conduct complex tasks that humans are capable of performing. AI is useful in the field of pathology, which involves analyzing images of the microscopic structure of different tissues. However, AI can be difficult to set up and apply to the task. One specific task, segmentation, involves picking specific structures out of tissue images and is a prime candidate for automation with AI. In our study, we have created a tool for pathology image segmentation which runs in the cloud (is accessible over the web). We demonstrate the tool by using it to segment various structures from kidney tissue. Our experiments show that the tool is easy to use, accurate, and can estimate the presence of one type of scarring as reliably as human experts.

## Abstract (web summary)

Lutnick et al. develop a cloud-based deep learning tool for whole slide image segmentation. The authors provide several examples of its application in renal pathology, for segmenting glomeruli, interstitial fibrosis and other features of interest.

## Introduction

Recent advances in machine learning techniques have led to previously unachievable performance for image analysis tasks. In particular, convolutional neural networks (CNNs)^1^, a form of deep learning, have great potential for impactful applications in the computational analysis of image structures. Successful adoption of these tools to biomedical image data promises a paradigm shift in both biological science and healthcare^2^.

In the field of pathology, the practice of digitizing histological slides has become common practice^3^, facilitating the application of CNNs for analysis. Digitally scanned histology slides, known as whole slide images (WSIs), are often gigapixels in size. Parsing WSIs into biologically relevant sub-compartments (commonly known as segmentation) is often an important first step for tissue analysis and pathological examination^4^. Due to the size of WSIs and the diversity of structures that can be present, downstream machine learning tasks (such as slide classification) can also benefit from segmentation, which can help limit the regions of interest considered^5^.

CNNs have been successfully utilized by many research groups for the segmentation of WSIs^4–9^. However, thus far tools to segment WSIs have been complex to deploy and use, requiring knowledge of the command line interface and computational expertize^10–12^. The ideal user for these tools is the pathologist or biological scientist, whose clinical workflow or research questions could benefit from fast and accurate segmentation of relevant structures^2^.

To address this gap, we have developed Histo-Cloud, a powerful tool for the segmentation of WSIs and deployed it as a suite of easy-to-use plugins using the Digital Slide Archive (DSA)^13^, an open-source cloud-based WSI repository with a built-in slide viewer. Histo-Cloud was designed with flexibility in mind and is agnostic to tissue type or structure. Segmentation of new structures of interest is possible by retraining the CNN used for segmentation, which can be conveniently performed within the cloud interface.

## Methods

Human data collection followed a protocol approved by the Institutional Review Board at University at Buffalo (STUDY00002731, STUDY00003929, STUDY00004044, STUDY00004235, STUDY00005089, and STUDY00005541) prior to commencement. Computational image analysis is done in this study using retrospective data qualified for a waiver of the consent process.

### WSIs for GlomTrainSet, GlomTestSet 1, and GlomTestSet 4

These datasets were used for the segmentation of glomeruli. This dataset consists of both human and murine renal tissue WSIs from various institutes as well as publicly available repositories, using diverse stains and different scanners. The institutions included the University of California at Davis (UC Davis), Johns Hopkins University (JHU), Kidney Translational Research Center (KTRC) at Washington University School of Medicine at St. Louis (WUSTL), Seoul National University Hospital Human Biobank (SNUHHB), Vanderbilt University Medical Center (VUMC), University at Buffalo (UB), University Hospital Cologne (UHC), and the publicly available Genotype-Tissue Expression (GTEx) portal, a repository that hosts human autopsy WSIs.

The GlomTrainSet consisted of 743 WSIs, 428 from humans and 315 from murine tissues, containing a total of 61,734 manually verified glomerular annotations. GlomTestSet 1 consisted of 100 holdout slides from the same data sources as GlomTrainSet. This included 3816 glomeruli, 37.8 GB of compressed image data, and a combined total of more than 0.24 trillion image pixels. GlomTestSet 4 contained an additional 1528 WSIs from the same sources that were used to study the scalability and prediction time of the method.

The human renal tissues manifest disease pathology spanning various stages of diabetic nephropathy; various classes of lupus nephritis; renal transplant protocol biopsies, including time-zero, protocol, and indication biopsy cases; human autopsy renal tissues publicly available via GTEx with diversity in age, sex, and race; and renal biopsies with pathologies that include membranous nephropathy, thrombotic microangiopathy, pauci-immune glomerulonephritis, focal segmental glomerulosclerosis (FSGS), mesangiopathic glomerulonephritis, arteriolosclerosis, hypertension, IgA nephropathy, chronic tubulointerstitial nephritis, acute tubular necrosis, Fabry disease, amyloid nephropathy, membranoproliferative glomerulonephritis, light chain cast nephropathy, minimal change disease, post-infectious glomerulonephritis, idiopathic nodular glomerulosclerosis, and anti-glomerular basement membrane disease. The human data were collected in accordance with protocols approved by Institutional Review Board at the UC Davis, JHU, KTRC, WUSTL, SNUHHB, VUMC, and UB. The SNUHHB data were shared under IRB number H-1812-159-998.

Murine renal tissues included in GlomTrainSet and GlomTestSet 1 came from three different models. For the first model wild-type, FVB/N mice were subjected to a combination of four interventions that induce a post-adaptive form of FSGS. The interventional process includes 0.9% saline drinking water, angiotensin II infused via an osmotic pump, uni-nephrectomy, and deoxycorticosterone delivered by implantation of a subcutaneous pellet, summarized as the SAND model^14,15^. The second model was a streptozotocin (STZ) diabetes murine model that manifests nephropathy; a detailed description of this model is discussed in our prior work^16^. The third model was a nephrin knockdown (nephrin KD) murine model, was implemented using a published protocol^17^, and shows mesangial hypercellularity and sclerosis, glomerular basement membrane thickening, and podocyte loss.

The tissues were sectioned at 2–5 µm thickness for staining and imaging. The data consist of tissues stained with diverse histological stains, including hematoxylin & eosin (H&E), periodic acid-Schiff (PAS) with hematoxylin (PAS-H) counterstain, Silver, Trichrome, Verhoeff’s Van Gieson, Jones, and Congo red. The slides were scanned using different brightfield microscopy WSI scanners, including Aperio VERSA digital whole slide scanner (Leica Biosystems, Buffalo Grove, IL), Nanozoomer (Hamamatsu, Shizuoka, Japan), and MoticEasyScan Pro (Motic, San Antonio, TX), at 40X resolution. The pixel resolution of the images used was 0.13 to 0.25 µm.

### WSIs for VessTrainSet, VessTestSet, and GlomTestSet 2

This human dataset was used to test the adaptability of the model for vessels. In total there were 939 annotated arteries, 6023 arterioles, and 4507 glomeruli. VessTrainSet contained 226 renal tissue WSIs. VessTestSet contained an additional 58 holdout slides. Multiple stains per case were used. This dataset was manually annotated for relevant structures to establish a ground-truth.

The renal tissue WSIs came from UHC via co-author J.U.B. Diagnoses included thrombotic microangiopathy, hypertension-associated nephropathy, and vasculitis. Tissues were sectioned at 2–3 µm thickness. Diverse histologic stains were used, including H&E, PAS-H, Masson trichome, and Jones methenamine silver, for staining the tissue to depict different pathobiological features. A brightfield microscopy scanner Nanozoomer (Hamamatsu, Shizuoka, Japan) was used for WSI scanning at 40X resolution. The pixel resolution of the images used was 0.25 µm. Note that the VessTestSet dataset was used to construct the GlomTestSet 2 dataset to conduct the study discussed in Glomeruli segmentation—scalability.

### WSIs for IFTASet 1, IFTASet 2, IFTASet 3, IFTATestSet 2, and GlomTestSet 3

These datasets were used for the segmentation of IFTA. The human renal tissues for this part of the study came from four institutions: the University of California, Davis; the University of California, Los Angeles (UCLA); University of Coimbra (Portugal); and University Hospital Cologne (UHC).

Tissues were obtained from renal allograft nephropathy with no prior history of rejection. For this study, periodic acid-Schiff (PAS)-stained renal tissue WSIs of renal allograft nephropathy were used for training (IFTASet 1, *n* = 20; IFTASet 2, *n* = 48; and IFTASet 3, *n* = 22). One slide was selected per case for each institution. The WSIs per set were uniformly chosen from four IFTA classes defined based on semiquantitative scores (ci/ct scores: 0, 1, 2, and 3); ci/ct scoring is a method defined in Banff 2018 criteria^18^ for assessing IFTA in transplant biopsies. A minimum of five slides per class were used for each set. The cases were reviewed to ensure the following selection criteria were met: (1) the amount of early or evolving IFTA with variable intermixed edema was minimized, (2) no active inflammation, (3) no prior history of rejection, and (4) cases were selected to represent the full range of IFTA severity. All types of IFTA, including classic, endocrinization, and thyroidization patterns, were included in the analysis, without distinguishing between the types. IFTATestSet 2 was provided by UHC, and contained 17 WSIs. This dataset followed similar case selection criteria as above with two slides from class 0 and five slides each from the remaining three classes.

The human data were collected in accordance with protocols approved by Institutional Review Boards at the UC Davis, UCLA, University of Coimbra, and the University at Buffalo. Deidentified images from UHC throughout this paper were used for retrospective research, and such is permitted under German law to conduct without IRB approval. The tissues were sectioned at 2–3 µm thickness and stained using PAS-H. Imaging was done using different brightfield microscopy WSI scanners, including Aperio CS virtual slide imaging system, Aperio AT2 (Leica Biosystems, Buffalo Grove, IL), and Nanozoomer (Hamamatsu, Shizuoka, Japan) at 40X resolution. Pixel resolution of the images used was 0.25 µm. Note that the IFTATestSet 2 dataset was used to construct the GlomTestSet 3 dataset to conduct the study discussed in Glomeruli segmentation—scalability.

### KPMP WSI dataset

This dataset was used to test the adaptability of the model for IFTA. This part of the study used 26 renal tissue biopsy whole slide images (WSIs) of 26 chronic kidney disease (CKD) subjects from the Kidney Precision Medicine Project. The selection of these slides followed the same criteria described in the section above: WSIs for IFTASet 1, IFTASet 2, IFTASet 3, IFTATestSet 2, and GlomTestSet 3. The recruitment sites were Brigham & Women’s Hospital, Cleveland Clinic, Joslin Diabetes Center/ Beth Israel Deaconess Medical Center, and the University of Texas at Southwestern. The inclusion criteria for CKD subjects for biopsy include subjects diagnosed with diabetic kidney disease (type 1 or 2) and hypertensive kidney disease. For the former, the subjects are included based on eGFR in the range of 30–59 mL/min/1.73 m^2^ or eGFR ≥ 60 with urinary protein to creatinine ratio (uPCR) >150 mg/g or urinary albumin to creatinine ratio (uACR) >30 mg/g. For the latter, the subjects are included based on eGFR in the range of 30–59 mL/min/1.73 m^2^ or eGFR ≥ 60 with uPCR in the range of 150–2000 mg/g or uACR in the range of 30–2000 mg/g. The study is overseen by three independent bodies, including a data safety monitoring board, a central institutional review board (WUSTL), and an NIH-NIDDK convened the external expert panel. More details about the rationale and design of KPMP cases are available in a recent publication^19^. The tissues were sectioned at 2–3 µm thickness, and the PAS-H stained tissues were used for the study presented in this work. Imaging was done using an Aperio GT450 brightfield microscopy WSI scanner (Leica Biosystems, Buffalo Grove, IL) at 40X resolution. The pixel resolution of the images used was 0.25 µm.

### WSIs for murine kidney tissue for the study discussed in murine model analysis—utility

For this part of study three murine model renal tissue WSIs were employed. These models include an aging model, and two type 2 diabetic nephropathy (T2DN) models (KKAy and Db/Db). We used eight mice (four young and four old) WSIs for the aging model, 20 mice (ten KKAy or disease and ten C57/BL6 or control) WSIs for the KKAy model, and 14 mice (7 Db/Db or disease and 7 Db/m or wild-type control) WSIs for the Db/Db model.

The aging studies were performed in 4-month-old and 21-month-old C57/BL6 male mice obtained from the NIA aging rodent colony^20^. For the KKAy model (see published description^21^), male mice that develop spontaneous diabetes of polygenic origin were used. For the Db/Db model, male mice with a BKS background featuring a leptin receptor mutation were used. These mice depict spontaneous/congenital diabetes due to leptin signaling abnormalities^22^. Animal studies were performed in accordance with protocols approved by the Institutional Animal Care and Use Committee at the Georgetown University, National Institutes of Health, JHU, and UB, are consistent with federal guidelines and regulations, and are in accordance with recommendations of the American Veterinary Medical Association guidelines on euthanasia. Tissues were sectioned at 2–3 µm thickness, and the PAS-H was used for staining. The slides were scanned using different brightfield microscopy WSI scanners, including Nanozoomer (Hamamatsu, Shizuoka, Japan) and MoticEasyScan Pro (Motic, San Antonio, TX), at 40X resolution. The pixel resolution of the images used was 0.25 µm.

### Software

**Fig. 1:**
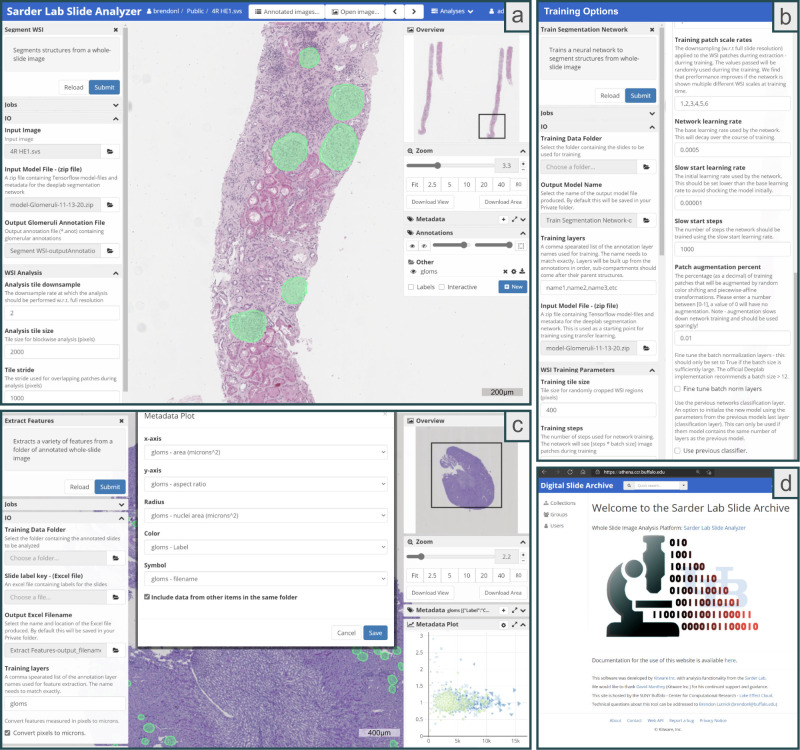
The user interface of the segmentation tool (available via the web). **a** The left <Segment WSI > column shows the controls for the segmentation plugin: <IO> is required arguments and <WSI Analysis> contains optional parameters. WSI stands for whole slide image and IO stands for Input/Output. The right column shows the WSI viewer controls and annotations created by the plugin. The green annotations are computationally predicted and are easily editable by the user. Slides are analyzed by clicking the <Submit> button in the top left corner. **b** The options from the <Train Segmentation Network> plugin. Under the <IO> section, a user can specify a directory full of annotated WSIs to use for network training with the <Training Data Folder> option, and where to save the trained model with the <Output Model Name> option. The <Training layers> option gives users the ability to choose which annotation layers should be used for training and multi-class segmentation models can be trained. To speed up the training process, a previously trained segmentation model can be used for transfer learning by specifying the <Input Model File>. Hyperparameters for training the network is automatically set to defaults that work well but can be modified using the options in the <WSI Training Parameters> section. **c** shows the <Extract Features> plugin which can be used to extract image and morphology features from annotated objects. These features are written to the slide metadata and can be plotted from within the online interface via the <Metadata Plot> tab (on the right). **d** shows the welcome screen of the online interface athena.ccr.buffalo.edu.

With the goal of developing a tool with class-leading WSI segmentation accuracy as well as easy accessibility to computational non-experts, we have integrated the popular semantic segmentation network Deeplab V3+^23^ with the DSA^13^, an open-source cloud-based histology management program. Specifically, we have created a suite of easy-to-use plugins using HistomicsUI, an application programming interface of the DSA for running Python codes. These plugins efficiently run the DeepLab network for native segmentation of WSIs, making testing new slides accessible through the HistomicsUI graphical user interface (the slide-viewing component of the DSA). Using the HistomicsUI interface, users can interactively view the computational annotations, and further refine such annotations for training new models. The modified HistomicsTK-Deeplab codebase is available via GitHub and also as a pre-built Docker image for easy installation. This software is deployed in the cloud and is accessible via the web, making it easily accessible to the community as a plug-and-play tool (Fig. 1). The open-source plugins are available to the digital pathology community for use and further development.

### Functionality

We have developed several plugin tools with various functions. (1) The <Segment WSI> plugin (Fig. 1a) segments WSIs using a previously trained model. (2) The <TrainNetwork> plugin can be used to train new models from a folder of annotated WSIs (Fig. 1b). Histo-Cloud generates predictions as a series of image contours or sparse heatmaps which are written to JavaScript Object Notation (JSON) format for display in HistomicsUI as annotation layers. The code is modular, with the ability to handle multi-class segmentation, and includes the option to tweak the network hyperparameters for advanced users. We include the ability to ignore image regions (Supp. Fig. 5), this is useful to exclude ambiguous image regions from the training set, and may also be of interest for users who wish to only annotate part of a large WSI. During training and testing, a progress bar is shown so the user can gauge the time to completion (Supp. Fig. 5). (3) Functionality was included for conversion between JSON annotations and the XML format (<IngestAperioXML> and <ExportAperioXML> plugins). The XML format is used to display contours in Aperio ImageScope (Leica, Buffalo Grove, IL) which is a popular WSI viewer. (4) The <ExtractFeaturesFromAnnotations> plugin (see Fig. 1c) was built for extraction of image and contour-based features from annotated regions in the slides. The features are written into the slide metadata (on DSA) in JSON format. For further data exploration, features saved into the slide metadata can be plotted pairwise using a scatterplot tool available in HistomicsUI (Fig. 1c) for a single slide or across a folder of WSIs. Features can also be saved in spreadsheet format for local download and further analysis.

### Computational model

We used the official implementation of the Deeplab V3 + segmentation network^23^, modified to work natively on WSIs. This implementation was accomplished by adapting the way the network ingests data and extracting patches from WSIs as needed during training using the large_image Python library^24^. A similar method (HistoFetch) is described more extensively in a recently published preprint^25^, which shows on-the-fly patch extraction speeds and overall training time for unsupervised tasks. The HistoFetch method was adapted in this work to perform a supervised segmentation task by creating additional patch selection criteria intended to proactively balance uneven class distributions during patch extraction. Note that during development the code was migrated to use large_image^24^ for reading WSI data rather than the openslide^26^ library, as the former supports a larger number of slide formats. To convert the ground-truth annotations to masks for semantic segmentation, the HistomicsUI JSON annotations are converted into the Aperio ImageScope XML format, and the XML_to_mask conversion code from the original H-AI-L study^7^ was reused for generating ground-truth masks. This code follows the way openslide and large_image read WSI patches via specifying the location and scale of the patches. The min and max indices of each contour annotation are written into the metadata of the XML, allowing for faster reference of which contours are in the image region requested.

A flowchart providing an overview of this training input pipeline is presented in Supp. Fig. 1. A similar pipeline is used during prediction (segmentation of slides), but patches are extracted deterministically from an overlapping grid pattern (excluding non-tissue regions) to ensure full tissue segmentation. The training and testing perform fast color thresholding of the tissue region which is saved as a portable network graphics (PNG) mask for reference (to avoid repeated operations). This process ensures the network does not train on non-tissue regions, and thus speeds the prediction process. During the development, we found that occasionally providing the network with background (non-tissue) patches helped generalize the batch normalization parameters during training. We, therefore, implemented a parameter that defines the probability of selection of patches that may include the background region. Default of 0.1 was found to work well in generalizing the batch normalization layers.

### Iterative learning and annotation ingestion

In a previous study, we showed that the human-in-the-loop annotation strategy significantly reduces the annotation burden when developing a tissue segmentation model^7^. This strategy uses a model trained on a limited dataset to run inference on new slides, which are corrected by an annotator. We find that the correction of computational annotations is faster than fully annotating newly added data, reducing the amount of effort required to build a robust training set. Additionally, this strategy allows the annotator to constantly interact with the system, monitoring its performance, and selecting slides where the model struggles the most for incorporation into the training set.

Human-in-the-loop annotation is possible using Histo-cloud through alternating use of the training and testing plugins. Practically, we expect that most users will start an annotation project from scratch and have made using pretrained ImageNet weights the default behavior of the training plugin. However, if a user would like to import data annotated in another system or format, we have included the <IngestAperioXML> plugin (which is described in the Functionality section above). This plugin is capable of ingestion of data annotations in the Aperio XML format and could be used to incorporate additional externally annotated data.

If an advanced user wishes to convert previously annotated data into the XML format for ingestion into the system, we direct them to the mask_to_xml script: https://github.com/SarderLab/Histo-cloud/blob/main/histomicstk/deeplab/utils/mask_to_xml.py This script was developed for the conversion of rasterized annotations into the XML format and is used internally by Histo-Cloud for a display of network predictions in HistomicsUI. For advanced users who wish to upload and manage XML annotations from the command line interface, we have also included scripts which satisfy these requirements in the source code: https://github.com/SarderLab/Histo-cloud/tree/main/batch_upload_xmls_to_girder_client.

### Training and testing

Training of models was done on a server equipped with two Intel Xeon Silver 4114 (10 core) processors, with 64 GB RAM and dual Nvidia Quadro RTX 5000 graphical processing units (GPU) with 16 GB of video random access memory (VRAM). These resources allowed training with a batch size of 12 using image patches of size 512 × 512 pixels. A batch size of 12 is the minimum recommended for training the batch normalization parameters in the DeepLab implementation document. The Athena server (open for public use) has only one GPU with 8 GB of VRAM. We have therefore disabled training of the batch normalization parameters by default in the training plugin (which can be enabled in the advanced parameter section) and have set a default batch size of 2. All trained networks used a base learning rate of 1e^−3^ with polynomial decay using the momentum optimizer (momentum value = 0.9).

All models use the Xception 65 network backbone^23^, with DeepLab parameters atrous_rates = 6, 12, and 18, output_stride = 16, and decoder_output_stride = 4 for both training and prediction. The glomerulus model was trained for 400,000 steps and was initialized using the ImageNet model. The vessel segmentation models were trained for 100,000 steps, and the IFTA segmentation models were trained for 50,000 steps using the ImageNet model as a starting point for transfer learning. Details on the trained models are outlined in Table 1.

**Table 1:** Data used and models trained.

Tasks	Structures segmented	Models trained	Initialization for transfer learning	Training WSIs	Holdout test WSIs	Independent test WSIs	Training steps
Data and models							
**Glomeruli segmentation**	Glomeruli	*Glomerulus model*	*ImageNet*	743	100	58 (*GlomTestSet 2*)	400,000
						17 (*GlomTestSet 3*)	
**Vessel segmentation**	Glomeruli, Arterioles, Arteries		*Random model*	226	58	Qualitative assessment of publicly available GTEx tissue WSIs from multiple organs	100,000
			*GTEx model*				
			*Glomerulus model*				
			*ImageNet*				
**IFTA segmentation**	IFTA, Glomeruli	*Institution 1*	*ImageNet*	12	29	17 (*IFTATestSet 2*)	50,000
		*Institution 2*	*ImageNet*	24			
		*Institution 3*	*ImageNet*	12			
		*Combined 1/3rd*	*ImageNet*	16			
		*Combined full*	*ImageNet*	48			
						26 (*KPMPTestSet*)	
**Murine model feature analysis**	Glomeruli	Used *Glomerulus model* for segmentation				4 old, 4 young	0
						10 KKAy T2DN, 10 C57 control	
						7 Db/Db T2DN, 7 Db/M control	

Different segmentation tasks, corresponding trained models, segmented structures, an initial model used for transfer learning, whole slide images (WSIs) used for training, hold-out testing, independent testing, and training steps. We note that GlomTestSet 2 is the same as the vessel segmentation holdout dataset (58 WSIs). GlomTestSet 3 is also the same as IFTATestSet 2 (17 WSIs).

As part of the input pipeline, WSI patches can be extracted efficiently at downsampled resolutions. The patch downsample rate is user-specified, and multiple downsample rates can be specified during training, which are randomly cycled for patch extraction. For training, downsample rates of 1, 2, 3, and 4 with respect to the native slide resolution were used, a randomly selected downsample rate from the list was used for each extracted training patch. For prediction, a downsample rate of 2 was used for all experiments, we found this choice was a good compromise between prediction speed and accuracy. We believe that the multi-resolution training strategy helped the network to generalize. We found the glomerulus model works equally well in both 40X and 20X WSIs (both using a prediction downsample of 2). Further, the vessel segmentation model was trained using 40X WSIs, and successfully applied to the 20X GTEx WSIs for testing.

Using a large patch size for prediction increased segmentation performance, giving the network a larger field of view and reducing-edge artifacts. For practical purposes, we settled on a default patch size of 2000 × 2000 pixels. For prediction, it was found that using a stride of 1000 pixels gave sufficient overlap between extracted patches. During prediction, the indices of the extracted patches are tracked, and the resulting bitmap prediction is used to populate a full WSI mask using the similar method as discussed in the original H-AI-L study^7^. To reduce the number of artifacts at the edge of the predicted patches, a parameter to remove the border of the predictions was included. Practically this parameter was set to remove 100 pixels from the border of each prediction.

To improve speed and to keep the memory requirements of code implementation low, network predictions are not up-sampled. Instead, the coordinates of the extracted contours or heatmap indices are up-sampled prior to JSON creation. Using DeepLab parameters, namely, output_stride = 16 and decoder_output_stride = 4, result in a prediction bitmap that is 25% of the size of the input resolution. With a default downsample of 2 used for prediction, the resultant WSI mask is one-eighth of the size of the pixel resolution of the original WSI. We found that 32 GB of RAM is enough to successfully segment even very large slides.

When experimenting with the network logits for the generation of the ROC plots (Fig. 4a, b), we converted the code to stitch the patch predictions together by averaging the logits of overlapping patches.

### Statistical analysis

Intraclass correlation coefficient measure (ICC)^27,28^ was used for the study shown in Fig. 4c, and corresponding r with null hypothesis *r* = 0 vs alternative *r* > 0 was used to measure significance. The ICC values were calculated using two-way random effects, absolute agreement, and single rater/measurement.

### Reporting summary

Further information on research design is available in the Nature Research Reporting Summary linked to this article.

## Results

To demonstrate Histo-Cloud’s performance characteristics and segmentation potential, a variety of segmentation tasks from renal biopsy WSIs were tested. For each task, performance was evaluated on holdout WSIs and independent test slides selected from datasets never used for training. A description of the datasets used for the studies below, including sources, disease pathology, tissue thickness, staining, and image acquisition is available in the Methods section and is summarized in Table 1. A list of abbreviations is listed in Supp. Table 1.

### Histo-cloud

Using the simple cloud-based interface, users can upload WSIs and train a segmentation network using their own annotations (see Fig. 1b). Users can iteratively apply Histo-Cloud’s training and prediction plugins in an active learning framework, to build up powerful segmentation models with reduced effort^7^. The segmentations produced by Histo-Cloud are converted to contours or heatmaps for direct display on the WSIs. When developing new segmentation models, the slide-viewing environment of this tool enables rapid qualitative evaluation of algorithm progress by displaying the network predictions (Fig. 1a).

Going beyond segmentation, an included modular plugin extracts features from segmented WSI tissue regions. These features are written into the metadata of uploaded slides and can be exported in spreadsheet form for further analysis. We have included a plotting tool in the user interface of the online slide viewer for quick exploration of these extracted features, Fig. 1c.

The source code can be run traditionally via the command line, but we expect the majority of users will utilize the intuitive HistomicsUI-based cloud interface (Fig. 1d). The source code is available on GitHub at https://github.com/SarderLab/Histo-cloud and packaged as a pre-built Docker image^29^ https://hub.docker.com/r/sarderlab/histo-cloud. This data sharing allows for easy deployment on a remote server for use as well as further development by the community over the web. Additionally, a publicly available instance of Histo-Cloud is available for the community at: athena.ccr.buffalo.edu. All the models described are available in the <Collections> section in the <Segmentation models> folder on athena.ccr.buffalo.edu or at https://bit.ly/3ejZhab. Documentation for using this tool is available at https://bit.ly/3nNMpfH. A video overview of Histo-Cloud is available at https://bit.ly/3r5GrZr.

### Glomerular segmentation—scalability

**Fig. 2:**
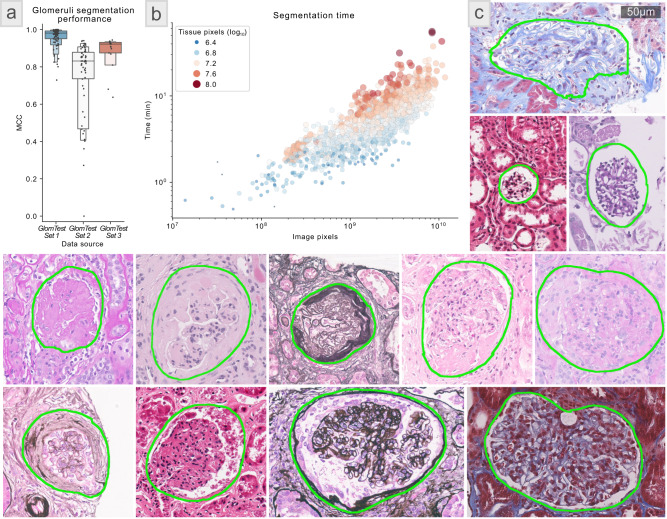
Glomeruli segmentation results—scalability study. **a** The segmentation performance of glomerulus model for glomeruli detection. Matthews correlation coefficients were calculated for three renal tissue whole slide image (WSI) datasets, as specified in subsection Glomeruli segmentation—scalability under the section Results. GlomTestSet 1 contained 100 WSIs holdout from the training set GlomTrainSet, GlomTestSet 2 had 58 WSIs, and GlomTestSet 3 had 17 WSIs. Both GlomTestSet 2 and GlomTestSet 3 were from an institution independent of the institutions from where the training dataset GlomTrainSet was formed for training the glomerulus model. Further, glomerular boundaries in GlomTestSet 2 and GlomTestSet 3 were annotated by an independent annotator who was not involved in annotating glomeruli in GlomTrainSet. Each dot represents a WSI. Box plot elements: The plot starts with the median as the centerline. Each successive level outward contains half of the remaining data. Namely, the first two sections out from the centerline contain 50% of the data. After that, the next two sections contain 25% of the data. This continues until we are at the outlier level. Each level out is shaded lighter. We used around 5–8 outliers in each tail. **b** shows the prediction time in minutes as a function of the WSI size in pixels for glomeruli predictions on 1528 WSIs in GlomTestSet 4. The color and size of the points represent the size of the automatically extracted tissue region of the slide (the analyzed region) in pixels. The proposed glomerular segmentation model scales roughly linearly in time for increasing WSI size. Each dot represents a WSI. **c** A batch of randomly selected glomeruli with the computationally segmented boundaries from the 100 holdout WSIs in GlomTestSet 1. This selection is intended to highlight the diversity of pathology and staining of the holdout dataset. The scale bar is 50 µm.

To assess the computational scalability of Histo-Cloud during training, a network model for glomeruli segmentation (glomerulus model) was trained using a very large dataset of renal tissue WSIs, containing 743 WSIs (GlomTrainSet). In total GlomTrainSet contained 1.8 trillion image pixels. Network performance was evaluated on a holdout set of 100 additional human renal tissue WSIs (GlomTestSet 1). The computationally generated segmentation was robust when compared with manual annotations for glomeruli and generated the following statistics: *F*-score = 0.97, Matthews correlation coefficient (MCC) = 0.97, Cohen’s kappa = 0.97, intersection over union (IoU) = 0.94, sensitivity = 0.95, specificity = 1.0, precision = 0.99, and accuracy = 1.0. This model also performed robustly on two independent test WSI datasets (GlomTestSet 2 and 3) originating from an institution not included in the training dataset with ground-truth established by a separate annotator (MCC = 0.83 and 0.90 on GlomTestSets 2 and 3, respectively) (Fig. 2a). Figure 2c shows examples of glomerulus segmentation performance for a diverse set of glomerular pathologic changes and histochemical stains.

We have found the performance of Histo-Cloud continually improves while achieving high specificity when deployed in a human-in-the-loop setting, using the method described in our previous work H-AI-L^7^. This process allows experts to iteratively correct the network predictions on holdout WSIs before incorporating them into the training set, and the subsequent training reduces future annotation burden^7^. This process is facilitated due to the ability of our system to view predictions interactively on the WSIs via the web interface, which is helpful to determine WSIs where the trained model struggles. We used this strategy to train the glomerulus model iteratively and obtained a decreasing number of incorrect segmentations with increasing iterations.

As part of the scalability study, the segmentation speed was assessed. Prediction time as a function of WSI size was tracked on a set of 1528 WSIs (median time = 4.7 min, median size = 1.9 Gigapixels) from a set that have similar diversity as in GlomTrainSet, we refer to this set as GlomTestSet 4. Histo-Cloud uses hardware acceleration on the host server to speed processing and can segment a large histology section in as little as 1 min. The segmentation time depends (approximately linearly) on the size of the tissue section; Fig. 2b quantifies segmentation speed as a function of image pixels on WSIs from GlomTestSet 4. The algorithm performs fast thresholding of the tissue region within the slide to reduce the computational burden for slides with large non-tissue areas. There is a slight programmatical overhead when opening, caching, and streaming data from larger slides, this appears as a gentle upslope of points of the same color in Fig. 2b.

### Vessel segmentation—adaptability

To evaluate the adaptability of Histo-Cloud for segmenting multiple structures from WSIs, we retrained the glomerulus model to segment glomeruli, arterioles, and arteries. The training set is referred to as VessTrainSet, and the test set is VessTestSet.

Transfer learning is a machine learning technique where a model developed for one purpose is retrained for another purpose^30^. Using the glomerulus model as the starting point for transfer learning, MCC of 0.91, 0.66, and 0.84 were obtained for segmenting glomeruli, arterioles, and arteries, respectively. The *MCC* metric was computed based on a pixel-wise agreement between computational segmentation and manual ground-truth. To study the effect of transfer learning on segmentation performance, we trained another model by randomly initializing the network parameters (random model); performance decreased to MCC of 0.55, 0.22, and 0.54, respectively, in segmenting the compartments.

We further explored the possibility of improving the computational performance without access to a model trained from a large segmented dataset. Toward this goal, we used the Genotype-Tissue Expression dataset (GTEx)^31^, which contains 15,989 H&E stained WSIs from 40 different tissue types, to pre-train a segmentation model to detect the tissue type. This was accomplished without any human annotation, by thresholding the tissue region of each slide and training a model to classify the tissue type of each slide. The goal was to create a model for transfer learning which had been exposed to diverse tissue morphologies, and therefore had learned filters useful for more fine-grained segmentation tasks. While transfer learning using the resulting model (GTEx model) did improve the segmentation performance of glomeruli, arteries, and arterioles (MCC = 0.77, 0.44, and 0.62, respectively) over random initialization, performance was below that achieved using the glomerulus model.

**Fig. 3:**
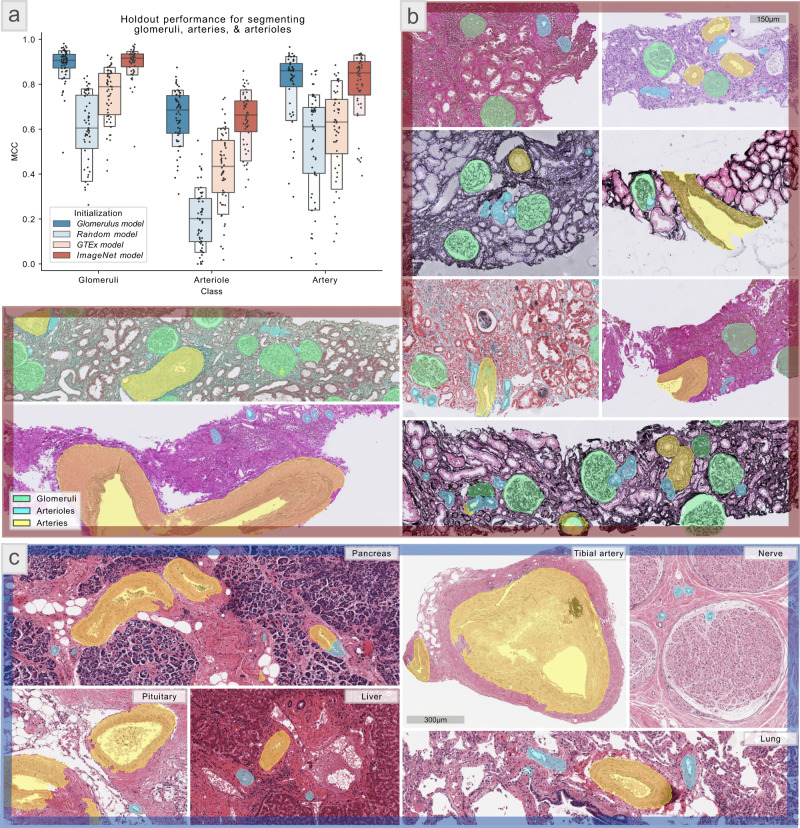
Vessel segmentation results—transfer learning study. **a** Segmentation performance as a function of network initialization (measured as Matthews correlation coefficient [*MCC*]) for the VessTestSet (58 holdout WSIs). The ground-truth annotations of structures were generated for segmenting three classes: glomeruli, arterioles, and arteries. The colors represent different transfer learning sources for parameter initialization. Namely, the glomerulus model is the model originally used for glomerular segmentation results in Fig. 2, offering *MCC* = 0.91, 0.66, and 0.84 for segmenting glomeruli, arteriole, and arteries, respectively. The random model does not use transfer learning for parameter initialization, offering *MCC* = 0.55, 0.22, and 0.54 in segmenting the three respective compartments. GTEx (genotype-tissue expression) model is a model originally trained to identify the diverse tissue types from the publicly available GTEx tissue WSI dataset (15,989 WSIs with 40 different tissue types), offering *MCC* = 0.77, 0.44, and 0.62 for the segmenting three respective compartments after transfer learning. ImageNet model uses a model pretrained on the ImageNet dataset, offering *MCC* = 0.91, 0.66, and 0.86 in segmenting the three respective compartments. Each dot in the box plot represents a WSI. Box plot elements: The plot starts with the median as the centerline. Each successive level outward contains half of the remaining data. Namely, the first two sections out from the centerline contain 50% of the data. After that, the next two sections contain 25% of the data. This continues until we are at the outlier level. Each level out is shaded lighter. We used around 5–8 outliers in each tail. **b** shows randomly selected crops of WSIs from the holdout set (VessTestSet) with computational segmentations by the model trained based on the ImageNet model as the starting point. The scale bar is 150 µm. **c** shows randomly selected crops of various types of tissues from GTEx WSIs, computationally segmented using the model trained based on the ImageNet model. Despite being trained only on kidney tissues, the trained model is able to segment arteries and arterioles in diverse tissue types. We also note that the GTEx slides are autopsy tissues scanned at 20X, and the training set for this study VessTrainSet was scanned at 40X, and did not contain autopsy tissue WSIs. The scale bar is 300 µm.

Finally, we trained a fourth model, transfer learning with a model pretrained on the ImageNet^32^ dataset, this same model was originally used to train the glomerulus model. Surprisingly, this model (ImageNet model) achieved the segmentation performance comparable to the glomerulus model (MCC = 0.91, 0.66, and 0.86, respectively). A more detailed comparison of these results is shown in Fig. 3a, with randomly selected holdout predictions from VessTestSet in Fig. 3b. To explore the performance of the ImageNet model on an independent test set, we segmented GTEx WSIs from different organs, examples are shown in Fig. 3c.

### Interstitial fibrosis and tubular atrophy (IFTA) segmentation—adaptability

To further evaluate the adaptability of Histo-Cloud, the effect of dataset variability on the segmentation of IFTA was studied in a distributed setting; namely, our web-based setup (in cloud). IFTA is morphological changes in the renal cortex reflecting “chronic” injury with resultant scar formation and is an important indicator to predict renal disease prognosis^9^.

To generate a ground-truth, three pathologists provided WSIs from their institutions and manually annotated IFTA. Past studies have shown significant disagreement among pathologists in manually annotating IFTA^9^. To minimize such disagreement, the pathologists used the definition of IFTA based on Banff 2018 criteria^18^, and also collaborated via our web-based tool in a distributed setup for IFTA annotation. Further, the inclusion criteria of cases (discussed in the Methods—WSIs from IFTASet 1, IFTASet 2, IFTASet 3, IFTATestSet 2, and GlomTestSet 3 section) minimized the variability of the annotation process.

A holdout dataset was randomly selected by pooling one-third of the slides from each institution (*n* = 29). We refer to this set as IFTATestSet 1. Another dataset from a fourth institution (IFTATestSet 2, *n* = 17) was used for independent testing. A pathologist from this fourth institution manually annotated IFTA in IFTATestSet 2 to generate the ground-truth.

We trained five models for IFTA segmentation using the pathologist-provided ground-truth: the first three models were trained using slides from a single institution—IFTASet 1 (12 slides), IFTASet 2 (24 slides), and IFTASet 3 (12 slides). We refer to these as Institution 1, 2, and 3 models respectively. The fourth model used the combined training data from all the three sets (48 slides), referred to as Combined full. A final model used 1/3rd of this combined set (16 WSIs), ensuring the amount of training data was comparable to the first three models. This model is referred to as Combined 1/3rd.

**Fig. 4:**
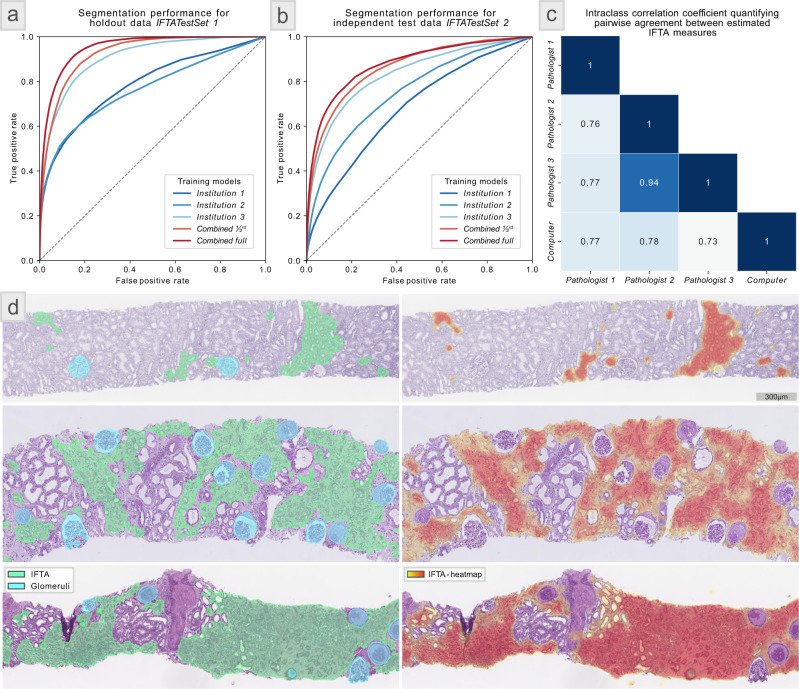
Interstitial fibrosis and tubular atrophy (IFTA) segmentation results—multi-institute study. **a** Receiver operating characteristic (ROC) plots showing the segmentation performance of five trained IFTA models on 29 holdout whole slide images (WSIs), IFTATestSet 1. Models—Institution 1, Institution 2, and Institution 3 were trained using datasets from three different institutions (with 12, 24, and 12 WSIs respectively). The Combined full model was trained by pooling these three datasets (48 WSIs). The Combined 1/3rd model used 1/3rd of the pooled training set, randomly selected (16 WSIs). This last model yielded better IFTA segmentation performance than the first three models, highlighting the importance of dataset diversity. The combined full model offered slightly better performance than the Combined 1/3rd model. **b** shows the performance of the five models on the independent test dataset IFTATestSet 2 with 17 WSIs. This dataset originated from an independent institution than those used in [**a**] and was annotated by an independent annotator. We observed the same performance trend as in [**a**]. **c** shows the pairwise Intraclass correlation coefficients (*ICC*) (*p* value < 0.05) for percent IFTA scored visually by three additional annotators and estimated based on computational segmentation using the Combined full model (computer) for the 26 WSIs in KPMPTestSet. The kidney precision medicine project (KPMP) cohort acted as another independent test set which was never seen by our trained model. **d** shows computational IFTA predictions using the Combined full model on the holdout WSIs IFTATestSet 1. The left shows the traditional contour predictions, the right shows the corresponding heatmap predictions developed specifically for structures with poorly defined boundaries.

To better assess the performance of the trained models, we output the network logits (predictions prior to using the argmax function) which were used to construct ROC plots for each model. This process allowed us to display IFTA predictions as heatmaps in HistomicsUI (Fig. 4d). Interestingly on IFTATestSet 1 training with 1/3rd of the combined dataset (Combined 1/3rd model) yielded better IFTA segmentation (AUC = 0.93) than training with a single institution dataset alone (Fig. 4a) (AUC = 0.78, 0.76, and 0.91 for models Institutions 1, 2, and 3, respectively). When we tested the Combined full model, the performance improved to AUC = 0.95. The same trend was observed when segmenting IFTA in the independent test set IFTATestSet 2 (Fig. 4b), with AUC = 0.68, 0.75, and 0.83 for models Institution 1, 2, and 3, respectively, AUC = 0.86 for Combined 1/3rd model, and AUC = 0.88 for Combined full model.

The IFTA segmentation models were trained to simultaneously segment IFTA and glomeruli. We observed the same performance trend for glomerulus segmentation via the IFTA models in both IFTATestSet 1 and 2; these results are available in Supp. Fig. 2. The ROC plots (generated by thresholding the network logits) for all the glomeruli, artery, and arteriole segmentations conducted in this work are shown in Supp. Fig. 5.

To demonstrate the robustness in another independent cohort and compare the trained model to a visual manual estimation of IFTA done in the clinical setting, we used an additional 26 PAS-stained chronic kidney disease renal biopsy cases from the Kidney Precision Medicine Project (KPMP)^33^ consortium. We refer to this set as KPMPTestSet. Three KPMP pathologists, provided a percent IFTA score to the nearest 10 percent for each slide following Banff 2018 definitions^18^. This scoring was done via visual estimation, without any annotation on the slides. The five IFTA segmentation models discussed above were used to segment IFTA boundaries in the KPMPTestSet, percent IFTA was estimated as segmented IFTA area over total renal cortex area, and the resulting computationally estimated scores were correlated with the manual visual estimation. Figure 4c shows a confusion matrix describing intraclass correlation coefficients (*p* value < 0.05) between pathologists and the computer for the Combined full model. We found that the correlation measures among pathologists and the computer models were excellent as per the convention provided by ref. ^34^, and thus is comparable. Supp. Fig. 4 shows a full comparison of the five IFTA segmentation models and each KPMP pathologist, the raw data for this calculation is available in Supp. Table 2. Figure 4d depicts examples of qualitative IFTA segmentation performance.

### Murine model analysis—utility

Finally, we show the utility of Histo-Cloud in a basic research application, analyzing digital image features extracted from computationally segmented glomeruli (via the Glomerulus model) from three murine models. A description of the models used is available in Methods—WSI from murine kidney tissue.

**Fig. 5:**
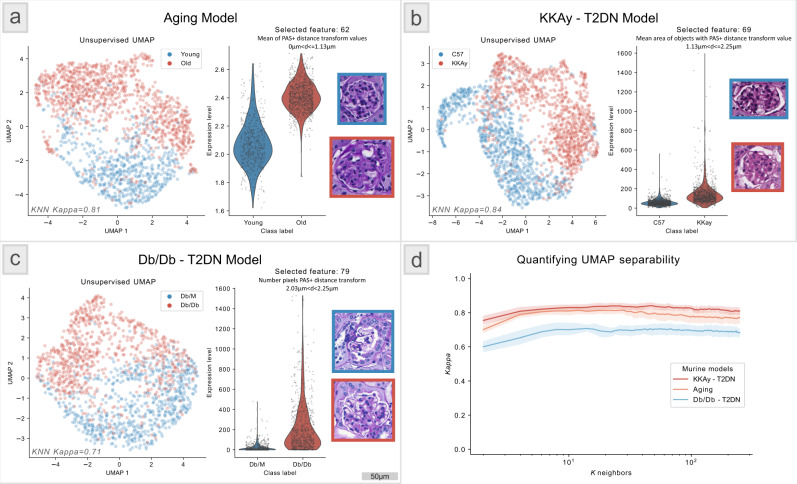
Murine model glomerulus feature analysis—utility study. Feature analysis from glomeruli segmented from renal tissue whole slide images (WSIs) from three murine models: **a** is an aging model and **b**, **c** are two type 2 diabetic nephropathy (DN) models (KKAy and Db/Db). In each panel, the left plot shows an unsupervised uniform manifold approximation and projection for dimension reduction (UMAP) representations of 315 engineered image features extracted from the murine glomeruli, where the glomeruli were segmented using the glomerulus model. Here each dot is a glomerulus and the red and blue colors differentiate the disease from the control. Definitions and quantification strategy of the 315 engineered image features are available in our prior work^5^. The right plot shows the highest differentially expressed feature as predicted using the Seurat software^37^. The representative glomeruli from each murine class depicting this differentially expressed feature, and the feature value, are shown on the right for each murine model. Each dot in the UMAP and violin plots in [**a**–**c**] represents a WSI. **d** shows a *K*-nearest neighbors (KNN) classifier performance plotting the Cohen’s Kappa measure as a function of *K* neighbors for classifying the unsupervised UMAP features with respect to disease vs control status for the murine models. This analysis was done using tenfold cross-validation using a similar method as formalized in a previous work^35^. Definitions of the 315 features are provided in Supp. Table 2. This study suggests that the seamless segmentation of glomeruli from large WSIs using our tool facilitates conducting deep glomerular feature analysis to study novel murine models.

WSI from each model contained multiple sections obtained from one murine, with an average of 90–200 glomeruli per section. For the current analysis, we extracted 315 engineered image features from each segmented glomerulus. Feature definitions and quantification methods are discussed in our prior work^5^, a description is also available in Supp. Data 7. The features were selected to reflect active, present, and physical manifestations of kidney pathophysiology. We used an unsupervised uniform manifold approximation and projection (UMAP)^35^ to learn a two-dimensional manifold in the feature space (performing dimensionality reduction). Each glomerulus was plotted (with label) in this space to visualize the separability between classes (control vs disease) in each murine model (Fig. 5). To quantify this separability, we trained a *K*-nearest neighbor (KNN)^36^ classifier using the UMAP features with fivefold cross-validation and computed the optimal Cohen’s kappa achieved over multiple *K* for each murine model (Fig. 5d). Overall, we found the aging, KKAy, and Db/Db diabetes models to have good unsupervised class separability (Fig. 5a–c). We also applied Seurat^37^ software to analyze the image feature data and to characterize differential feature abundance. The distribution of the top feature separating control from disease, and the most representative glomeruli image patches depicting differences between these two classes are shown in Fig. 5.

## Discussion

In this work, we contribute three elements to the digital pathology community to advance tissue analysis: an online tool, the source code, and trained segmentation networks. We believe that easy-to-use AI tools and collaborative development of powerful models will benefit the digital pathology research community.

This work was motivated by our previously developed Human-AI-Loop (H-AI-L)^7^ which allows for iterative annotation of WSIs significantly reducing the annotation burden. As most work in computational pathology, H-AI-L has found limited utilization by the pathology research community due to the complexities of installation. To address this limitation, we implemented Histo-Cloud as an online tool which does not require the installation of any software on the user’s local computer. All processing occurs on the remote server, which hosts the web client. Like the original H-AI-L work, we use the DeepLab segmentation network^23^ for processing image patches, but Histo-Cloud uses on-the-fly processing of WSI patches, streaming data directly from the slides to increase the tool’s performance and scalability. Data permissions (set via the digital slide archive—DSA^13^) can be adjusted to keep uploaded data secure.

Annotation done interactively on the WSI fits easily into pathologist workflow, and the cloud-based nature of Histo-Cloud abstracts any computational overhead away from the end-user. Annotation can be done on any internet-connected device without any software installation. If the user prefers to annotate locally, we have added options to ingest and export annotations in an extensible markup language (XML)^38^ format readable by the commonly used WSI viewer Aperio ImageScope^39^. The authors note two complimentary works: HistomicsML^40^ and Quick Annotator^41^, both use superpixels^42^ and active learning^43^ to speed the annotation process. HistomicsML also uses HistomicsUI for deployment, and Quick Annotator is run locally in the QuPath slide viewer^44^. A future extension of our tool will combine edge detection and snapping^45^ to speed up the initial segmentation by human annotators.

Conducting the transfer learning study using the GTEx tissue histology WSIs (Fig. 3a) (15,989 WSIs containing 2.6 trillion total image pixels, 4.7 TB of data) and training the glomerulus model for glomeruli segmentation (Fig. 2a) (743 WSIs, 1.8 trillion pixels, 276 GB) were stress tests for scalability. Setting Histo-Cloud’s accessibility benefits aside, the study of glomeruli segmentation (Fig. 2) not only uses the largest most-diverse cohort of WSIs, but also reports the best performance in the literature for glomerular segmentation. In our previous work on H-AI-L^7^ we trained Deeplab-v2^46^ using a dataset of 13 PAS and hematoxylin and eosin (H&E) stained murine WSIs containing 913 glomeruli, and achieved an *F*-score = 0.92. Kannan et al.^47^ used Inception-V3^48^ for the sliding window classification of glomeruli with a set of 885 patches from 275 trichrome-stained biopsies and reported MCC = 0.63. Bueno et al.^49^ trained U-net^6^ with 47 PAS-stained WSIs and reported Accuracy = 0.98. Gadermayr et al.^50^ used 24 PAS-stained murine WSIs to train U-net^6^, reporting Precision = 0.97 and Sensitivity = 0.86.

Jayapandian et al.^51^ present the most comprehensive results on glomeruli segmentation, training U-net^6^ on a dataset containing 1196 glomeruli from 459 human WSIs stained with H&E, PAS, Silver, and Trichrome, reporting *F*-score = 0.94. However, their analysis is limited to glomeruli with minimal change disease^52^. In contrast, our training dataset (GlomTrainSet) contained a large dataset of 743 WSIs from both humans and mice, stained with diverse histological stains, with 61,734 total glomeruli, from diverse disease pathologies beyond minimal glomerular changes. The holdout dataset GlomTestSet 1 contained similar diversity (Fig. 2c). Our trained model also performed well on independent test datasets GlomTestSet 2 and 3 (Fig. 2a). Predictably, performance on GlomTestSet 2 and 3 (which contain slides from institutions never seen during training) was lower than the holdout dataset. Despite this, a visual assessment of the independent test set segmentation by expert pathologists was favorable. The modularity of Histo-Cloud will allow others to adapt the trained model to include more structurally abnormal glomeruli.

When testing the effectiveness of transfer learning, we found that adapting the ImageNet model for segmenting glomeruli, arteries, and arterioles using the VesselTrainSet, performed equivalently to using the glomerulus model as the starting point. The ImageNet model was trained on thousands of natural image classes and is widely used in computer vision literature as a generalized feature extractor^32^. It is surprising that despite having refined its convolutional features on renal tissue the glomerulus model did not offer a performance improvement for another renal tissue segmentation task. This result suggests that it may be better to start network training using the ImageNet parameters which offer a very generalized set of features more applicable to the segmentation of any tissue type (this is now the default for training Histo-Cloud models in the cloud). Encouragingly, when applying the developed vessel segmentation model to different tissue types from the publicly available GTEx tissue WSIs^31^, the segmentation of arteries and arterioles was found to be consistent with expert opinion (Fig. 3c).

Perhaps the most interesting aspect of a cloud-based segmentation tool is the ease of crowdsourcing annotation and developing collaborative models across centers or institutions^53^. As discussed above and also known that manual annotation of IFTA boundaries by multiple pathologists suffer from a high degree of disagreement^9^. In contrast, Histo-Cloud’s web-based system allowed the annotators to view each other’s annotations in annotating IFTASet 1, 2, and 3, and IFTATestSet 2 for the multi-institute IFTA study (see IFTA segmentation—adaptability under Results). We further note that visualizing IFTA prediction confidence using heatmaps was more reflective of the underlying biology than using contours, confirmed by subject matter experts via visual assessment. Namely, a heatmap depicts a probability, which is more informative than contours, which display binary predictions. Examples of IFTA segmentations on the holdout data IFTATestSet 2 as both contours and heatmaps are shown in Fig. 4d. The functionality to output segmented regions as heatmaps is available using the segmentation plugin.

The IFTA segmentation study further highlights the importance of training set diversity. Training using data from more institutions improved segmentation performance, even when less WSIs from each institution were used. Namely the performance of the Combined 1/3rd model in comparison to Institution 1, Institution 2, and Institution 3 models (see IFTA segmentation—adaptability under Results). This and the results described in the previous paragraph suggest a cloud-based environment is ideal for the development of models for histology segmentation, avoiding bias and allowing easy interaction between annotators for generating ground-truth by centralizing data from multiple institutions. Users can choose to pool their data or simply utilize models trained by others to aid in annotation or for transfer learning.

Finally, the murine model analysis case study suggests that our tool will enable basic science laboratories working on murine experiments to study differential abundant image features in various disease models as well as in treatment groups. In summary, the analytic approaches described here will enable researchers who lack software engineering skills to analyze histopathology from murine models or human tissue, using an intuitive online cloud-based framework. In the future, we plan to extend the capabilities of Histo-cloud to include instance segmentation as well as classification of tissues.

## Supplementary information

Supplemental Material

Supplemental Data 1

Supplemental Data 2

Supplemental Data 3

Supplemental Data 4

Supplemental Data 5

Supplemental Data 6

Supplemental Data 7

Supplemental Data 8

Description of Additional Supplementary Files

Peer Review File

Reporting Summary

## Data Availability

The digital pathology WSI data are in.svs or.scn format which uses lossless compression to represent the information content in images in pyramidal form. Images used in this work can be accessed based on shared data from our earlier publications; namely, from https://bit.ly/3PmcO1F^5^, https://bit.ly/3eywm0J^9^, https://bit.ly/3e6XZzs^54^, and https://goo.gl/cFVxjn^7^. Further, the dataset from the KPMP consortium is openly available via https://www.kpmp.org/available-data. The KPMP renal tissue biopsy WSI database contains more than 1000 WSIs and can be used for validating as well as additional training of the computational tools developed in this article. Moreover, a running instance (Athena) of Histo-Cloud is available for public testing and select WSIs have been made available via this public instance. Links to these resources can be found in the Introduction—Histo-Cloud section. We also include Supp. Data 1–6 in.xlsx format to provide the source data used for generating graphs and plots in Figs. 2–5 as well as Supp. Figs. 2, 3, respectively. Other reasonable requests for data can be submitted to the corresponding author, and the data will be shared following local institutional regulatory requirements.

## Code availability

The source code can be run traditionally via the command line, but we expect most users will utilize the intuitive HistomicsUI-based cloud interface (Fig. 1d). The source code is available on GitHub at https://github.com/SarderLab/Histo-cloud and packaged as a pre-built Docker image^29^ https://hub.docker.com/r/sarderlab/histo-cloud. This data sharing allows for easy deployment on a remote server for use as well as further development by the community over the web. Additionally, a publicly available instance of Histo-Cloud is available for the community at: athena.ccr.buffalo.edu. All the models described are available in the <Collections> section in the <Segmentation models> folder on athena.ccr.buffalo.edu or at https://bit.ly/3ejZhab. Documentation for using this tool is available at https://bit.ly/3nNMpfH. A video overview of Histo-Cloud is available at https://bit.ly/3r5GrZr. The code at the time of publishing is archived on Zenodo^55^.
