# Supplementary material for: A user-friendly tool for cloud-based whole slide image segmentation with examples from renal histopathology
Source: Commun Med (Lond). 2022 Aug 19;2:105. doi: 10.1038/s43856-022-00138-z (PMC9391340; doi:10.1038/s43856-022-00138-z)
Supplement: Supplementary file 1 — Supplemental Material [file 43856_2022_138_MOESM1_ESM.pdf]

A user-friendly tool for cloud-based whole slide image segmentation with examples from renal histopathology – **Supplemental Material**

Brendon Lutnick[1], David Manthey[2], Jan U. Becker[3], Brandon Ginley[1], Katharina Moos[3], Jonathan E. Zuckerman[4], Luis Rodrigues[5], Alexander J. Gallan[6], Laura Barisoni[7], Charles E. Alpers[8], Xiaoxin X. Wang[9], Komuraiah Myakala[9], Bryce A. Jones[10], Moshe Levi[9], Jeffrey B. Kopp[11], Teruhiko Yoshida[11], Jarcy Zee[12], Seung Seok Han[13], Sanjay Jain[14], Avi Z. Rosenberg[15], Kuang Yu. Jen[16], Pinaki Sarder[1,], for the Kidney Precision Medicine Project[†]*

[1]Department of Pathology and Anatomical Sciences, SUNY Buffalo, Buffalo, USA
[2]Kitware Incorporated, Clifton Park, USA
[3]Institute of Pathology, University Hospital Cologne, Cologne, Germany
[4]Department of Pathology and Laboratory Medicine, University of California at Los Angeles, Los Angeles, USA
[5]University Clinic of Nephrology, Faculty of Medicine, University of Coimbra, Coimbra, Portugal
[6]Department of Pathology, Medical College of Wisconsin, Milwaukee, USA
[7]Departments of Pathology and Medicine, Duke University, Durham, USA
[8]Department of Laboratory Medicine and Pathology, University of Washington, Seattle, USA
[9]Departments of Biochemistry and Molecular & Cellular Biology, and [10]Department of Pharmacology and Physiology, Georgetown University, Washington, DC, USA
[11]Kidney Disease Section, NIDDK, NIH, Bethesda, USA
[12]Department of Biostatistics, Epidemiology, & Informatics, University of Pennsylvania, Philadelphia, USA
[13]Department of Internal Medicine, Seoul National University College of Medicine, Seoul, South Korea
[14]Department of Medicine, Nephrology, Washington University School of Medicine, St. Louis, USA
[15]Department of Pathology, Johns Hopkins University, Baltimore, USA
[16]Department of Pathology and Laboratory Medicine, University of California at Davis, Sacramento, USA

[†]A full list of members and their affiliations appears in Supp. Data 8

[*]Address all correspondence to: Pinaki Sarder
Tel: 716-829-2265; E-mail: pinakisa@buffalo.edu

SUPPLEMENTAL MATERIAL

Supplemental Figure 1. Flowchart of the custom DeepLab WSI input pipeline.

Supplemental Figure 2. Glomeruli segmentation performance using IFTA segmentation models.

Supplemental Figure 3. ROC performance for glomeruli and vessel segmentation.

Supplemental Figure 4. Correlation of percent IFTA estimation between methods.

Supplemental Figure 5. More Histo-Cloud interface features.

Supplemental Table 1. Table of abbreviations.

Supplemental Table 2. Raw percent IFTA scores.

SUPPLEMENTAL FIGURES

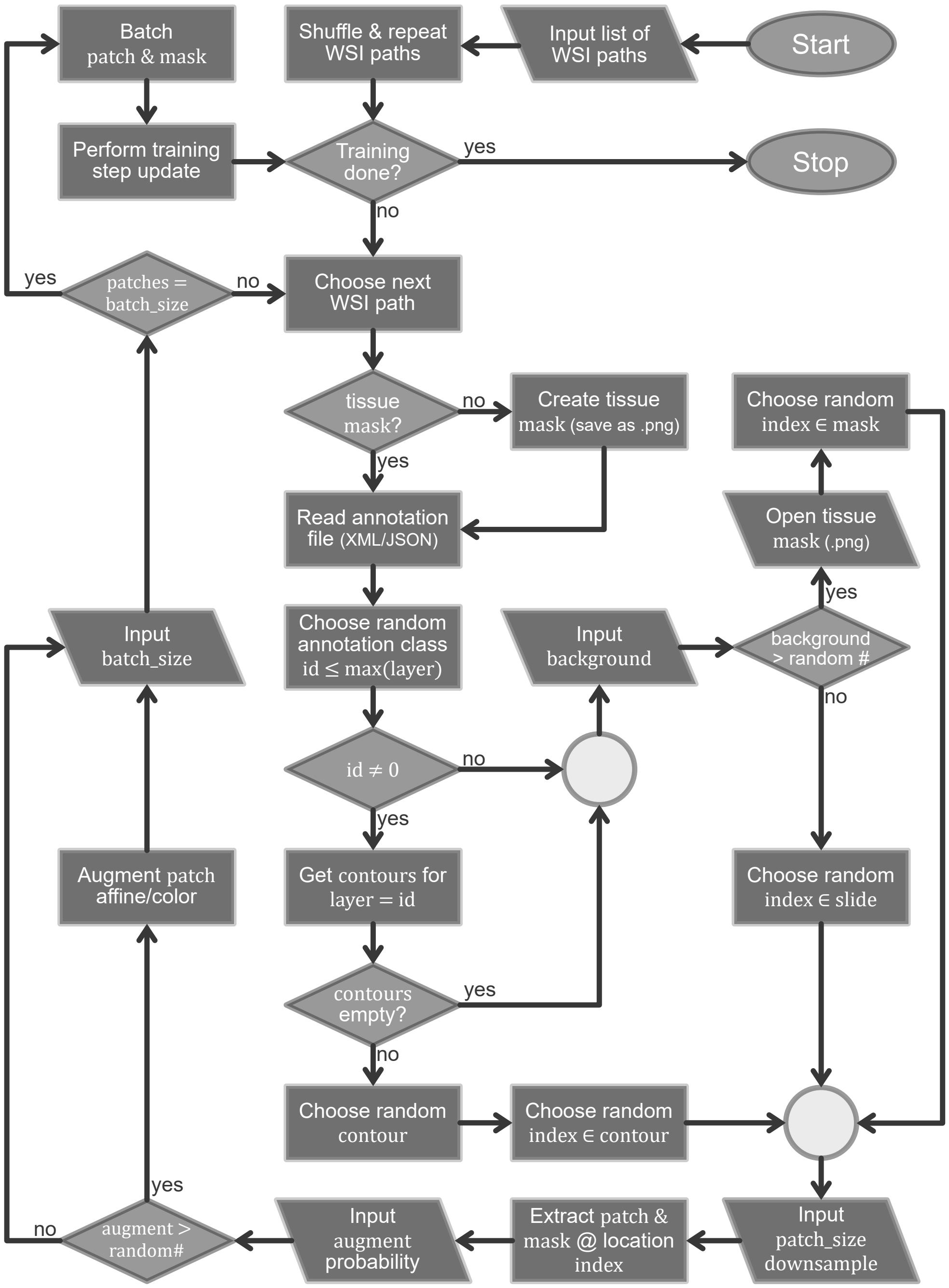

Supplemental Fig. 1 | Flowchart of the custom DeepLab WSI (whole slide image) input pipeline.

The details of the custom input pipeline used by our modified DeepLab code to ingest WSI data during training. The *large_image* python library (https://github.com/girder/large_image/) is used to extract patches from WSIs on-the-fly. This process uses a modified version of our *HistoFetch* pipeline[1], which has been further modified to work for supervised learning tasks. For the network training, pixel locations from the image data corresponding to each data class are randomly selected by exploiting the XML (extensible markup language) or JSON (JavaScript object notation) annotation files. This ensures class balancing for network training by selecting appropriately sampled pixel regions for all the classes. If the background class is selected, a random location within the tissue region (which has been pre-segmented via morphological processing) is selected. During application development, we found that occasionally providing the network with non-tissue patches as background helped the batch normalization parameters to generalize, which reduced error. We therefore added a parameter defining the probability of selection of a non-tissue region, allowing patches within and outside the tissue regions to be included in the analyses. When using a trained model to segment structures (prediction on new slides), a similar pipeline is used. However, image patches to be processed are extracted deterministically from an overlapping grid pattern (excluding non-tissue regions), to ensure the entire tissue region is processed for full segmentation. This input pipeline is predominantly implemented in the following files in the DeepLab codebase available via github: /datasets/wsi_data_generator.py and /utils/wsi_dataset_util_large_image.py

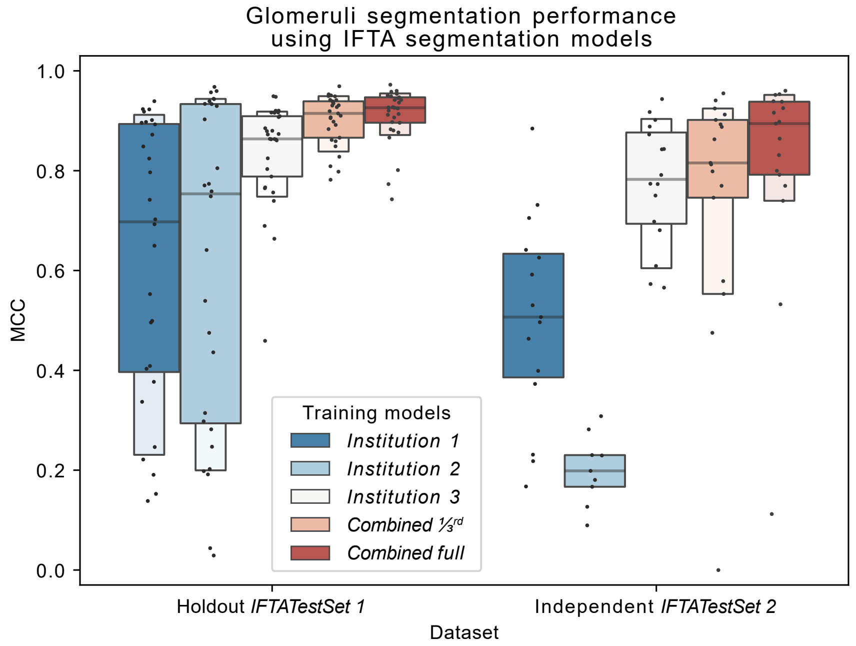

Supplemental Fig. 2 | Glomeruli segmentation performance using interstitial fibrosis & tubular atrophy (IFTA) segmentation models.

The glomerular segmentation performance using the five models trained for segmenting IFTA and glomeruli (see *IFTA SEGMENTATION – adaptability* under *RESULTS* and ***Fig. 4***). The performance is quantified on the *IFTATestSet 1* with 29 holdout renal tissue WSIs, and the independent test set *IFTATestSet 2* with 17 renal tissue WSIs (whole slide images) and annotation ground-truth originated from an institution independent of the training dataset and *IFTATestSet 1*. We observe the same trend in performance as IFTA segmentation as shown in ***Fig. 4***. Namely, the *Combined full* model delivers the best performance, while the *Combined 1/3*rd model performs better than any of the models trained on a single institution data alone. Here each dot represents the performance on one WSI. Box plot elements: The plot starts with the median as the centerline. Each successive level outward contains half of the remaining data. Namely, the first two sections out from the centerline contain 50% of the data. After that, the next two sections contain 25% of the data. This continues until we are at the outlier level. Each level out is shaded lighter. We used around 5-8 outliers in each tail.

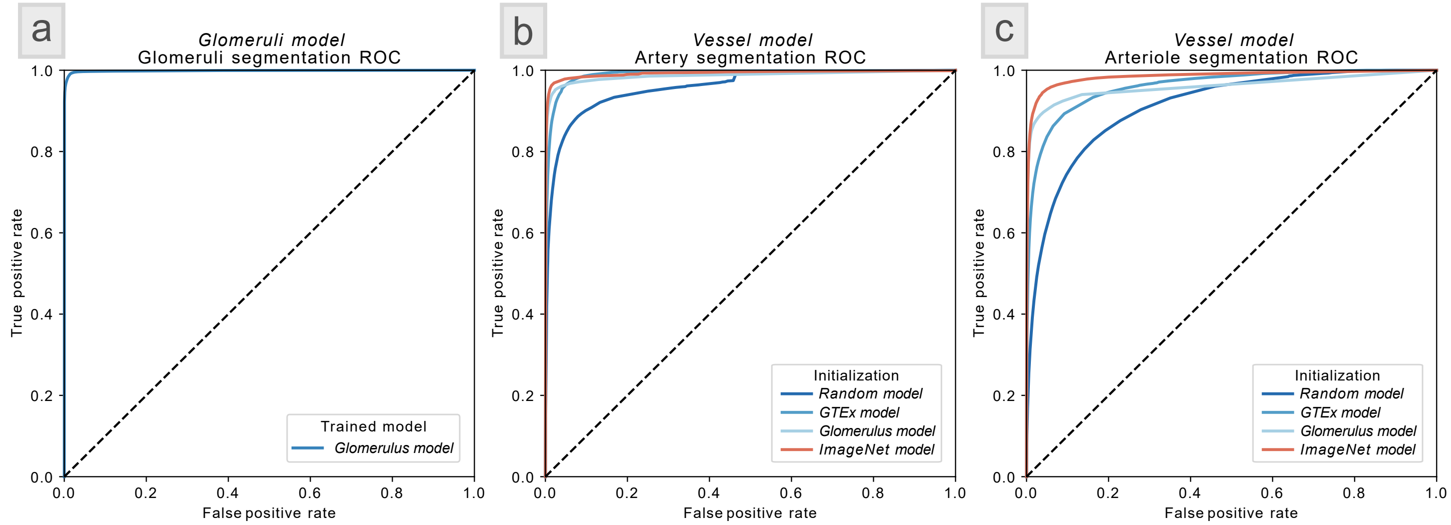

Supplemental Fig. 3 | ROC (receiver operating characteristic) performance for glomeruli and vessel segmentation.

Panel [a] shows the ROC performance for glomerular segmentation on *GlomTestSet 1* with 100 holdout renal tissue WSIs (whole slide images) using *glomerular model* (see *GLOMERULI SEGMENTATION – scalability* in *RESULTS* and ***Fig. 2***). **[b]** shows the ROC performance for artery segmentation on the holdout dataset *VessTestSet* with 58 renal tissue WSIs using the four initialization strategies for vessel segmentation (see *VESSEL SEGMENTATION – adaptability* in *RESULTS* and ***Fig. 3***). **[c]** shows the ROC performance for arteriole segmentation on the same holdout dataset with same initialization strategies as used in **[b]**.

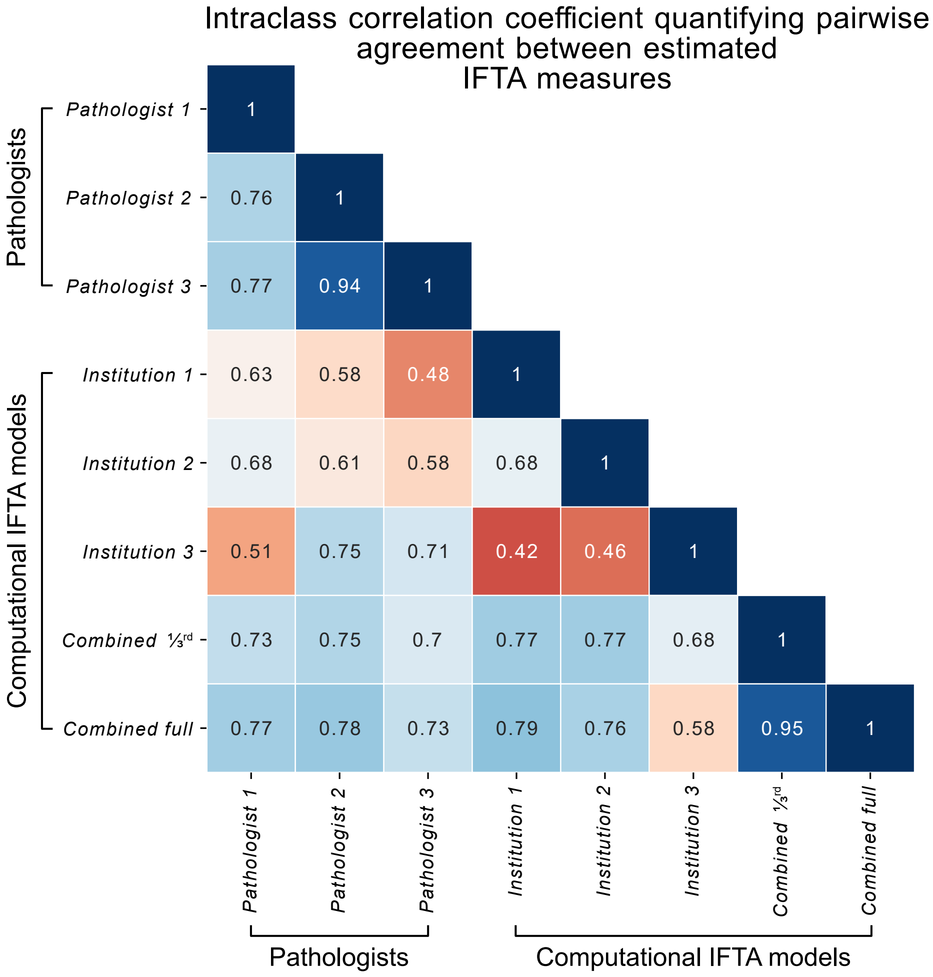

Supplemental Fig. 4 | Correlation of percent interstitial fibrosis & tubular atrophy (IFTA) estimation between methods.

Intraclass correlation coefficient (ICC) quantifying pairwise agreement between computationally and manually estimated IFTA measures for 26 chronic kidney disease (CKD) renal tissue WSIs (whole slide images) from Kidney Precision Medicine Project (KPMP) cohort data, *KPMPTestSet*. The ICC values were calculated using two-way random effects, absolute agreement, and single rater/measurement. Pairwise correlations were measured for each pair of the three KPMP pathologists and the five computational models trained for IFTA segmentation (see *IFTA SEGMENTATION – adaptability* under *RESULTS*). This result is an extension of ***Fig. 4c***. Intraclass correlation coefficients with p-value < 0.05 are shown.

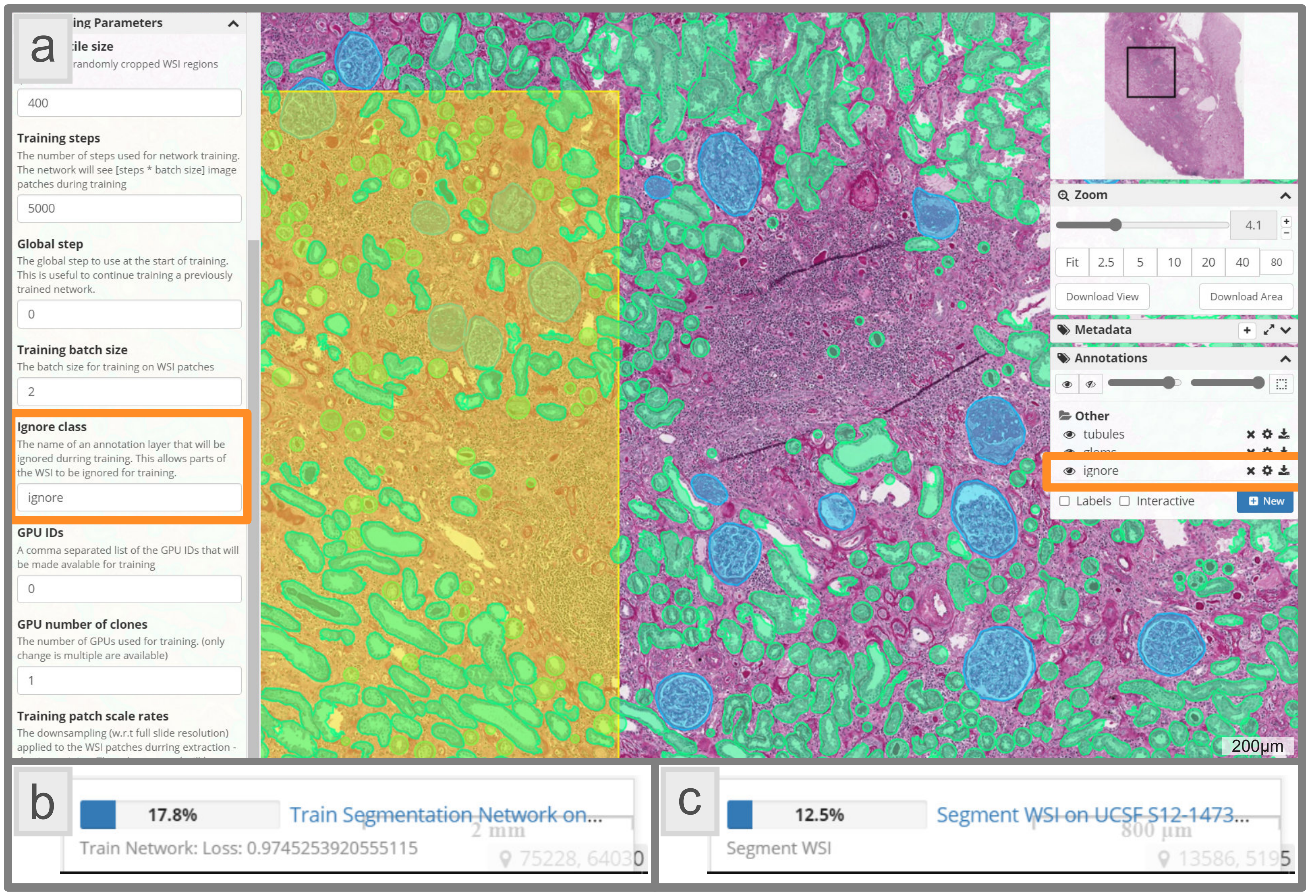

Supplemental Fig. 5 | More *Histo-cloud* interface features.

Panel [A] highlights the ability to ignore regions of the slide during training. This is done by annotating the regions users wish to be ignored (in this case the yellow rectangle), and specifying the name of this layer in the *Training Parameters* section of the training plugin. This feature can be useful for ambiguous regions, or for large slides where it is too much effort to fully annotate the entire WSI (whole slide image). **[B]** shows a progress bar that appears in the bottom right corner of the screen when a user submits a training job using the *TrainNetwork* plugin. This bar shows progress of the training job and highlights the network loss during training. **[C]** shows a similar progress bar that appears when running the *SegmentWSI* plugin.

TABLE OF ABBREVIATIONS

Supplementary Table 1 | Table of abbreviations.

AUC	Area under the curve
CKD	Chronic kidney disease
CNN	Convolutional neural network
DSA	Digital slide archive
eGFR	Estimated glomerular filtration rate
FSGS	Focal segmental glomerulosclerosis
GPU	Graphics processing unit
GTEx	Genotype-tissue expression
H-AI-L	Human – Artificial Intelligence – Loop
H&E	Hematoxylin and eosin (stain)
HIVAN	HIV-associated nephropathy
IFTA	Interstitial fibrosis and tubular atrophy
IoU	Intersection over Union (Jaccard index)
JSON	JavaScript object notation
KD	Knockdown
KPMP	Kidney precision medicine project
MCC	Matthews correlation coefficient
PAS	Periodic acid–Schiff (stain)
PNG	Portable network graphics
RAM	Random access memory
ROC	Receiver operating characteristic
STZ	Streptozotocin
T2DN	Type 2 diabetic nephropathy
uACR	Albumin to creatinine ratio
UMAP	Uniform manifold approximation and projection
uPCR	Urinary protein to creatinine ratio
VRAM	Video RAM
WSI	Whole slide image
XML	Extensible markup language

RAW IFTA SCORING DATA

Supplementary Table 2 | Raw percent IFTA scores.

The raw percent IFTA scores for the 26 KPMP WSIs. Pathologists estimated percent interstitial fibrosis & tubular atrophy (IFTA) to the nearest 10%. For the Intraclass correlation coefficient calculation in ***Fig. 4*** and ***Supp Fig. 4*** the percent IFTA scores obtained by the computational models were not rounded.

WSI	Pathologist 1	Pathologist 2	Pathologist 3	Institution 1	Institution 2	Institution 3	Combined 1/3rd	Combined Full
1	40	50	40	34.1	39.1	48	33.7	28.1
2	20	20	20	37.3	14.5	43.6	25.2	19.4
3	10	0	0	2.7	2	6.3	2.5	3.3
4	10	10	10	13.3	12	20.3	17.1	14.6
5	60	50	60	26.8	28.8	53.1	40.5	35.8
6	10	10	10	5.2	8.4	26.5	9.1	8.9
7	10	20	20	6.4	14.4	27.9	20.2	16.4
8	0	0	0	0.6	13.9	20.3	5.8	1.6
9	10	20	30	4.7	8.9	13.3	9	9.4
10	30	30	50	8	29.7	43.7	28.3	25.4
11	20	50	40	12.2	13.9	47.8	17.1	17.1
12	0	0	0	2.3	1.4	5.3	3.2	3.9
13	0	10	10	4.1	10	49.2	24.2	11.9
14	0	10	10	5.7	7.5	12.3	6.5	7.8
15	10	20	20	11	12.3	22.9	17.9	17.2
16	10	0	0	3.3	0.8	14.1	2.9	4.7
17	30	40	50	15.9	24	26.8	19.4	22.9
18	0	0	0	1.3	5.2	3.3	1.8	2.4
19	20	0	0	0.2	0	3.6	0.6	0.6
20	20	30	30	15.5	19.7	34.5	25.9	25.5
21	0	20	20	5.7	6.9	24.9	8.9	9.9
22	30	20	30	5.9	11.2	18.9	11.8	16.1
23	30	40	40	33.3	18.6	38.8	38.3	41
24	30	50	50	26.1	21.7	43.6	29.4	36.8
25	0	0	0	3.6	1.9	10	3.5	3.5
26	20	30	30	21.5	10.1	29.3	26.9	24.4

SUPPLEMENTAL REFERENCES

1 Lutnick, B., Krishna, L. M., Ginley, B. & Sarder, P. Histo-fetch -- On-the-fly processing of gigapixel whole slide images simplifies and speeds neural network training. *arXiv preprint arXiv:2102.11433* (2021).
