# Supplementary material for: A user-friendly tool for cloud-based whole slide image segmentation with examples from renal histopathology
Source: Commun Med (Lond). 2022 Aug 19;2:105. doi: 10.1038/s43856-022-00138-z (PMC9391340; doi:10.1038/s43856-022-00138-z)
Supplement: Supplementary file 10 — Description of Additional Supplementary Files [file 43856_2022_138_MOESM10_ESM.pdf]

Description of Additional Supplementary Files

File Name: Supplementary Data 1

Description: Tab 1. Data used to produce the glomeruli segmentation performance plot in Fig. 2a. Tab 2. Raw data from Fig. 2b.

File Name: Supplementary Data 2

Description: Raw data for creating Fig. 3a.

File Name: Supplementary Data 3

Description: Tab 1. Raw data for producing the Receiver operating characteristic plots in Fig. 4a. Tab 2. Raw data for producing the Receiver operating characteristic plots in Fig. 4b. Tab 3. The raw percent IFTA (interstitial fibrosis & tubular atrophy) scores used to calculate Intraclass correlation coefficient to derive the confusion matrix in Fig. 4c.

File Name: Supplementary Data 4

Description: Tab 1. Raw data from Fig. 5a. Tab 2. Raw data from Fig. 5b. Tab 3. Raw data from Fig. 5c. Tab 4. Raw data from Fig. 5d.

File Name: Supplementary Data 5

Description: Raw data for Supp. Fig. 2.

File Name: Supplementary Data 6

Description: Tab 1. Raw data for producing the ROC (receiver operating characteristic) plots in Supp. Fig. 3a. Tab 2. Raw data for producing the Receiver operating characteristic plots in Supp. Fig. 3b. Tab 3. Raw data for producing the Receiver operating characteristic plots in Supp. Fig. 3c.

File Name: Supplementary Data 7

Description: List of features measured on each glomerulus.

File Name: Supplementary Data 8

Description: Members of kidney precision medicine project (KPMP)
