# Supplementary material for: A user-friendly tool for cloud-based whole slide image segmentation with examples from renal histopathology
Source: Commun Med (Lond). 2022 Aug 19;2:105. doi: 10.1038/s43856-022-00138-z (PMC9391340; doi:10.1038/s43856-022-00138-z)
Supplement: Supplementary file 12 — Reporting Summary [file 43856_2022_138_MOESM12_ESM.pdf]

nature research

Corresponding author(s): Pinaki Sarder

Last updated by author(s): Mar 29, 2022

Reporting Summary

Nature Research wishes to improve the reproducibility of the work that we publish. This form provides structure for consistency and transparency in reporting. For further information on Nature Research policies, see our Editorial Policies and the Editorial Policy Checklist.

Statistics

For all statistical analyses, confirm that the following items are present in the figure legend, table legend, main text, or Methods section.

n/a	Confirmed	
☐	☒	The exact sample size (*n*) for each experimental group/condition, given as a discrete number and unit of measurement
☐	☒	A statement on whether measurements were taken from distinct samples or whether the same sample was measured repeatedly
☒	☐	The statistical test(s) used AND whether they are one- or two-sided<br>*Only common tests should be described solely by name; describe more complex techniques in the Methods section.*
☒	☐	A description of all covariates tested
☒	☐	A description of any assumptions or corrections, such as tests of normality and adjustment for multiple comparisons
☒	☐	A full description of the statistical parameters including central tendency (e.g. means) or other basic estimates (e.g. regression coefficient) AND variation (e.g. standard deviation) or associated estimates of uncertainty (e.g. confidence intervals)
☐	☒	For null hypothesis testing, the test statistic (e.g. *F*, *t*, *r*) with confidence intervals, effect sizes, degrees of freedom and *P* value noted<br>*Give P values as exact values whenever suitable.*
☒	☐	For Bayesian analysis, information on the choice of priors and Markov chain Monte Carlo settings
☒	☐	For hierarchical and complex designs, identification of the appropriate level for tests and full reporting of outcomes
☒	☐	Estimates of effect sizes (e.g. Cohen's *d*, Pearson's *r*), indicating how they were calculated

Our web collection on statistics for biologists contains articles on many of the points above.

Software and code

Policy information about availability of computer code

Data collection	No software was used
Data analysis	The source code can be run traditionally via the command line, but we expect the majority of users will utilize the intuitive HistomicsUI based cloud interface (Fig. 1d). The source code is available on GitHub at https://github.com/SarderLab/Histo-cloud, and packaged as a pre-built Docker image https://hub.docker.com/r/sarderlab/histo-cloud. This data sharing allows for easy deployment on a remote server for use as well as further development by the community over the web. Additionally, a publicly available instance of Histo-Cloud is available for the community at: athena.ccr.buffalo.edu. All the models described are available in the <Collections> section in the <Segmentation models> folder on athena.ccr.buffalo.edu or at https://bit.ly/3ejZhab. Documentation for using this tool is available at https://bit.ly/3nNMpfH. A video overview of Histo-Cloud is available at https://bit.ly/3r5GrZr.

For manuscripts utilizing custom algorithms or software that are central to the research but not yet described in published literature, software must be made available to editors and reviewers. We strongly encourage code deposition in a community repository (e.g. GitHub). See the Nature Research guidelines for submitting code & software for further information.

Data

Policy information about availability of data

All manuscripts must include a data availability statement. This statement should provide the following information, where applicable:

- Accession codes, unique identifiers, or web links for publicly available datasets
- A list of figures that have associated raw data
- A description of any restrictions on data availability

The digital pathology WSI data are in .svs or .scn format which uses lossless compression to represent the information content in images in pyramidal form. Images used in this work can be accessed based on shared data from our earlier publications; namely, from https://bit.ly/3PmcO1F54, https://bit.ly/3eywm0J55, https://

bit.ly/3e6XZzs56, and https://goo.gl/cFVxjn7. Further, the dataset from the KPMP consortium is openly available via https://www.kpmp.org/available-data. The KPMP renal tissue biopsy WSI database contains more than 1000 WSIs and can be used for validating as well as additional training of the computational tools developed in this article. Moreover, a running instance (Athena) of Histo-Cloud is available for public testing and select WSIs have been made available via this public instance. Links to these resources can be found in the Introduction – Histo-Cloud section. We also include Supp. Data 1-6 in .xlsx format to disburse the data used for generating graphs and plots in Fig. 2-5 as well as Supp. Fig. 2-3, respectively. Other reasonable requests for data can be submitted to the corresponding author, and the data will be shared following local institutional regulatory requirement.

Field-specific reporting

Please select the one below that is the best fit for your research. If you are not sure, read the appropriate sections before making your selection.

☒ Life sciences ☐ Behavioural & social sciences ☐ Ecological, evolutionary & environmental sciences

For a reference copy of the document with all sections, see nature.com/documents/nr-reporting-summary-flat.pdf

Life sciences study design

All studies must disclose on these points even when the disclosure is negative.

Sample size	This is a tool development study and we used largest available whole slide image repository from renal pathology for this study to train and test our models
Data exclusions	Not applicable. This is a tool development study.
Replication	Not applicable. This is a tool development study.
Randomization	The tool was tested via dividing the samples as training and test data following best practices in machine learning literature.
Blinding	The tool was tested via dividing the samples as training and test data following best practices in machine learning literature.

Reporting for specific materials, systems and methods

We require information from authors about some types of materials, experimental systems and methods used in many studies. Here, indicate whether each material, system or method listed is relevant to your study. If you are not sure if a list item applies to your research, read the appropriate section before selecting a response.

Materials & experimental systems

n/a	Involved in the study	
☒	☐	Antibodies
☒	☐	Eukaryotic cell lines
☒	☐	Palaeontology and archaeology
☒	☐	Animals and other organisms
☒	☐	Human research participants
☒	☐	Clinical data
☒	☐	Dual use research of concern

Methods

n/a	Involved in the study	
☒	☐	ChIP-seq
☒	☐	Flow cytometry
☒	☐	MRI-based neuroimaging
