# Supplementary material for: A user-friendly tool for cloud-based whole slide image segmentation with examples from renal histopathology
Source: Commun Med (Lond). 2022 Aug 19;2:105. doi: 10.1038/s43856-022-00138-z (PMC9391340; doi:10.1038/s43856-022-00138-z)
Supplement: Supplementary file 11 — Peer Review File [file 43856_2022_138_MOESM11_ESM.pdf]

nature portfolio

Peer Review File

A user-friendly tool for cloud-based whole slide image segmentation with examples from renal histopathology

Open Access This file is licensed under a Creative Commons Attribution 4.0 International License, which permits use, sharing, adaptation, distribution and reproduction in any medium or format, as long as you give appropriate credit to the original author(s) and the source, provide a link to the Creative Commons license, and indicate if changes were made. In the cases where the authors are anonymous, such as is the case for the reports of anonymous peer reviewers, author attribution should be to 'Anonymous Referee' followed by a clear attribution to the source work. The images or other third party material in this file are included in the article's Creative Commons license, unless indicated otherwise in a credit line to the material. If material is not included in the article's Creative Commons license and your intended use is not permitted by statutory regulation or exceeds the permitted use, you will need to obtain permission directly from the copyright holder. To view a copy of this license, visit http://creativecommons.org/licenses/by/4.0/.

Reviewers' comments:

Reviewer #1 (Remarks to the Author):

This is an integrated work compiling the author group's previous publications with some extensions and newly added experiments. Although not novel, this work does provide an accessible tool for researchers and pathologists who need to construct their own segmentation models on WSIs. The manuscript is generally well organized. However, several points need to be addressed.

1. Line 31. The authors proposed Histo-Cloud, a WSI segmentation tool. It would be good if the authors make some comparison with similar existing tools such as Aiforia Create.
2. Line 50. The authors stated that "Due to the size of these images and the diversity of structures that can be present, parsing WSIs into biologically relevant sub-compartments is often an important first step for tissue analysis." This is a misleading statement. Parsing WSIs into sub-compartments is not due to the size of WSI nor the diversity of structures, but because of the need of histological or pathological examination.
3. Line 104-115. A glomerulus segmentation model was trained to assess the computational scalability of Histo-Could. However, this paragraph mentioned only generalizability rather than scalability. The MCC dropped from 0.97 to 0.83 and 0.90 on two independent test datasets; are they statistically significant?
4. Line 116-123. This paragraph did not mention scalability either. The authors are suggested to re-write or abridge these paragraphs. The author stated that "We have found that the performance of Histo-Cloud continually improves while achieving high specificity when deployed in a human in the loop setting." but did not provide any data. Is it part of the study in this paper? Or it was done in author's previous papers. If so, references should be provided. In addition, the author should at least provide some data showing that the "loop" can be operated on the current Histo-Could. In addition, how much time will it take to go through a "loop" on Histo-Cloud?
5. Line 208-213. "…the resulting computationally estimated scores were correlated with the manual visual estimation." and Figure 4c and Supp. Figure 4. Pearson correlation coefficient is not a valid statistical method for this comparison. Pearson correlation coefficient only examine the correlation, but here the aim is the reproducibility of percent IFTA. Please consider intraclass correlation coefficient instead. If ICC is used, please do specify the chosen "Mode", "Type", and "Definition". Also, please provide raw data for the statistics in supplementals.
6. Line 263. It would be better if the authors mention the size of training data for glomerulus segmentation model in the first paragraph of Glomerular segmentation – scalability in RESULTS to emphasize it's a scalability test.
7. Line 280. "Predictably, performance on the independent test datasets GlomTestSet 2 & 3 was lower than the holdout dataset, albeit visual manual assessment of glomerular segmentations appeared to be consistent with expert opinion." Please clarify this sentence.
8. Line 284. Here in DISCUSSION and in VESSLE SEGMENTATION – adaptability in RESULTS (and in Figure 3a), both Cohen's kappa and MCC were used, which is quite confusing. Cohen's kappa was used in DISCUSSION and in the figure legend of Figure 3a (line 737-743), but the y-axis in Figure 3a showed MCC. Please consider using homogenous expression. Either Cohen's kappa or MCC showed very similar values between models constructed from Glomerulus-pretrained model or ImageNet-pretrained model. Therefore, "When testing the effectiveness of transfer learning, we found that adapting the glomerulus model for segmenting glomeruli, arteries, and arterioles using the VesselTrainSet, did not perform as well as when the same dataset was trained using the ImageNet model as the starting point (albeit very marginally)." might be an overstatement, unless the authors

could show that the results from two pretrained models are statistically different. The following discussion in the paragraph was based on the assumption that the ImageNet-pretrained model is truly better. It is advised that the authors re-write this paragraph.
9. Line 401. Did the KPMP WSI dataset for testing of ITFA model follow the selection criteria mentioned in Line 385-389?
10. Line 530. As mentioned, Pearson correlation coefficient is not a valid statistical method for the comparison.
11. Line 713. Figure 2b. Please use Tissue pixels for x-axis instead. It is more relevant to consider the size of “tissue” pixels rather than “whole image” pixels.

Reviewer #2 (Remarks to the Author):

The authors propose Histo-Cloud: a tool for segmentation of WSIs with an interactive graphical user interface. Histo-Cloud builds functionality on top of the HistomicsTK interface by enabling CNNs to segment regions within WSIs. The tool also enables features to be extracted from segmented regions for further analysis. The paper is well organised and is easy to follow and understand throughout.

Overall, the method is interesting, and it aims to reduce the effort needed for users to train segmentation-based models. The entry level required for developing deep learning models is lowered by packaging the software in the form of a GUI. This is good, but I would like to hear more on what controls are in place to ensure models are trained correctly. For example, pathologists may not annotate exhaustively, which is typically required for supervised segmentation. What happens in this case? I would also like to know more on what the GUI displays during model training. Are any statistics or plots shown? Also, can you add more information on how a user would connect the application to their own compute?

Is there an option to export the trained model? After all, a user may want to use the model in a downstream application, but not necessarily in Histo-Cloud. Also, it would be good if the user could add their own custom model rather than relying on the DeepLab v3 architecture. For example, a user may have their own developed model that performs well, but they want to improve the performance using Histo-Cloud. Why was TensorFlow selected? If using the application for improving model performance, then users may want to also explore PyTorch models – please give justification for the choice of deep learning library.

I would like more emphasis to be placed on the platform rather than the segmentation models. I understand that specific applications should be highlighted, but state with more clarity why Histo-Cloud enabled this.

How will Histo-cloud be extended? Why is it limited to just segmentation? Hypothetically this should not need to be limited to just segmentation.

Are there different options for visualisation? Such as the colour of overlay and transparency? Does the segmentation work on the entire WSI or on image regions within the WSI?

We thank the reviewers for the comprehensive and positive comments. We discuss below how we have addressed all the critiques/ concerns raised.

We also wanted to note the following:

1- *We have removed the HIVAN mouse analysis under Section "MURINE MODEL ANALYSIS - UTILITY" without losing the central theme of the paper. We left this model out as this model has not been published by our collaborators in its full potential and we accidentally included this result in the previous submission.*
2- *We have edited the manuscript throughout to improve the presentation in this revised version.*

Referee expertise:

Referee #1: Renal pathology

Referee #2: Computational pathology, deep learning

Reviewers' comments:

Reviewer #1 (Remarks to the Author):

This is an integrated work compiling the author group's previous publications with some extensions and newly added experiments. Although not novel, this work does provide an accessible tool for researchers and pathologists who need to construct their own segmentation models on WSIs. The manuscript is generally well organized. However, several points need to be addressed.

We thank Reviewer 1 for their kind words, and have addressed the points raised below individually. These revisions in the text are highlighted in Yellow.

1. Line 31. The authors proposed Histo-Cloud, a WSI segmentation tool. It would be good if the authors make some comparison with similar existing tools such as Aiforia Create.

We thank the reviewer for pointing out Aiforia Create. As an academic lab focused on developing computational tools for WSI analysis we do not have access to paid software such as Aiforia. We suspect that many researchers are in a similar situation and would greatly appreciate freely available and open-source tools such as Histo-Cloud.

That said, we did reach out to Aiforia who graciously provided a trial of their software so we could qualitatively evaluate the differences. This software would cost us $6000 per year as an academic lab, and this does not include the costs for compute time. While we have a demo of Histo-cloud available, users with very large datasets and compute requirements will be better served by setting up their own instance on AWS or their own hardware, potentially requiring some IT support.

Overall, the features offered by Aiforia seem very similar to Histo-Cloud, and instance segmentation is the only headline feature missing in Histo-Cloud. Being closed source, the model architecture used internally by Aiforia is not transparent to the end user, which we feel is an important omission, and will restrict downstream provenance which is an important goal for National Institutes of Health as part of their upcoming Bridge2AI program for generating AI ready datasets; see https://commonfund.nih.gov/bridge2ai.

Overall, the user experience of Histo-cloud and Aiforia are similar, but the two software tools are targeted at very different userbases. Histo-cloud is open-source and transparent, a tool that the digital pathology community can adopt and iterate to their unique needs. Aiforia is closed source and proprietary, and as a paid software comes with a warranty and support. Unlike Histo-cloud, models created in the Aiforia environment cannot be used in outside applications.

We have summarized the relevant differences in the following table:

Feature	Histo-cloud	Aiforia
WSI database	x	x
WSI viewer	x	x
Cloud based	x	x
No software install required	x	x
Segmentation training	x	x
Segmentation inference (prediction)	x	x
Works directly on WSIs	x	x
Open Source	x	
Transparent model architecture	x	
Trained models can be used outside tool	x	
Supports multi-class segmentation	x	x
Instance segmentation		x
Collaborative annotation	x	x
Polygon / freehand annotation	x	x
Prediction displayed as a heatmap	x	
Hardware acceleration	x	x
Ability for local hosting	x	
AWS compatibility	x	x
Partial slide annotation	x	x
Export annotations for local viewer	x	
Professional support		x

We wanted to note that integration of instance segmentation in Histo-Cloud is straightforward and is part of our plan for a future work. Further, we are going to receive funding to further develop Histo-Cloud, and as part of this work we will start a Slack channel for Histo-Cloud userbase, to adaptively improve the potential of Histo-Cloud with active feedback from the users.

Overall, the open-source nature of Histo-Cloud, and the modularity and scalability of the tool has potential to democratize and decentralize biomedical image analysis research. The recipe provided in this work will motivate developers to extend further on top of current development and allow the tool to be hosted in a server framework where users can search for pertinent data while bringing their own data for solving important problems in biomedicine. Finally, the extension of current features will allow standardization of computational models for downstream benchmarking and provenance. This direction will champion the goals of multi-disciplinary national consortiums, such as Kidney Precision Medicine Project consortium https://www.kpmp.org/ and Human BioMolecular Atlas Project (HuBMAP) consortium https://hubmapconsortium.org/ , where the ultimate goals are fusion of multi-modal and multi-omics data for solving important research questions in biomedicine. Such envisioned democratization and decentralization of biomedical data and tools will not be possible by using paid software where the source code is proprietary and business and profit get higher priorities over academic research.

2. Line 50. The authors stated that "Due to the size of these images and the diversity of structures that can be present, parsing WSIs into biologically relevant sub-compartments is often an important first step for tissue analysis." This is a misleading statement. Parsing WSIs into sub-compartments is not due to the size of WSI nor the diversity of structures, but because of the need of histological or pathological examination.

We thank the reviewer for pointing out this misleading statement. We agree that for clinical workflows segmentation is essential for pathological examination. We had intended to point out the benefits that segmentation can offer when attempting to perform downstream machine learning analysis (such as classification of the slide).

In many cases segmentation can reduce the amount of tissue in a slide that is considered for a classification task. This step simplifies the task by limiting the amount of tissue diversity that needs to be considered by an algorithm for the downstream classification. For example, in our previous work, Ginley et al., Journal of the American Society of Nephrology, 2019, https://jasn.asnjournals.org/content/30/10/1953.abstract , used computationally segmented glomeruli from renal tissue whole slide images for downstream classification of the WSIs to estimate the diabetic nephropathy class following the Tervaert classification scheme.

We have updated our phrasing of this point in the introduction, and hope that our intention is sufficiently clear.

3. Line 104-115. A glomerulus segmentation model was trained to assess the computational scalability of Histo-Could. However, this paragraph mentioned only generalizability rather than scalability. The MCC dropped from 0.97 to 0.83 and 0.90 on two independent test datasets; are they statistically significant?

*We have clarified our intention (i.e., why this shows scalability) – showing that we can train Histo-cloud using a very large dataset (743 WSIs). Scalability is further shown as part of the same section via evaluation using 1528 WSIs (GlomTestSet4); see the last paragraph of the subsection GLOMERULAR SEGMENTATION – scalability under the section RESULTS and **Fig. 2b**. We further discussed in Paragraphs 4 & 5 under DISCUSSION that the data we use for training the glomerular segmentation model is the largest in the literature further strengthening the scalability aspect of our model. All these relevant paragraphs clarified our intention for scalability in addition with generalizability.*

While the reviewer asks whether MCC values are statistically significant, we believe that conducting this hypothesis test is not appropriate for our research goals and therefore could be distracting or even misleading. A hypothesis test for a statistically significant difference between training and testing sets is highly dependent on sample size and variability within the datasets. Our objective was to develop models that can accurately segment histologic structures from renal biopsy images across different patient populations and sites and regardless of sample size. Since the MCC decreased in the external test datasets, the model may have learned too specifically in the training dataset. Still, the MCCs of 0.83 and 0.90 demonstrate that the glomerulus segmentation model performed well and was generalizable for independently collected datasets. Therefore, the magnitude of the MCCs is of greatest importance in our analysis. Further our authorship team contains eight renal pathologists, and all of them anonymously agreed via visual assessment that the segmented glomeruli in the two independent test datasets satisfactorily match their definition of glomeruli as used in histopathologic evaluation. We added this clarification in the DISCUSSION section Paragraph 5.

4. Line 116-123. This paragraph did not mention scalability either. The authors are suggested to re-write or abridge these paragraphs. The author stated that “We have found that the performance of Histo-Cloud continually improves while achieving high specificity when deployed in a human in the loop setting.” but did not provide any data. Is it part of the study in this paper? Or it was done in author’s previous papers. If so, references should be provided. In addition, the author should at least provide some data showing that the “loop” can be operated on the current Histo-Could. In addition, how much time will it take to go through a “loop” on Histo-Cloud?

We have clarified why this section is a scalability study in the previous paragraph.

We have also added clarification about the human in the loop training; see Paragraph 2 of the subsection GLOMERULAR SEGMENTATION – scalability under the section RESULTS. We have extensively quantified the benefits of human in the loop training and iterative annotation of training data in our previous work H-AI-L: https://www.nature.com/articles/s42256-019-0018-3. *While this paper was cited in this sentence, we see that this discussion was originally not clear enough.*

The intention of Histo-Cloud is not to reiterate the conclusions of the H-AI-L study but to demonstrate the utility and ease of use of the Histo-Cloud tool. However, the ability to view and correct computational predictions for performing human in the loop training is described in our documentation of the tool. We point the reviewer to the included documentation of Histo-Cloud (step 14): https://bit.ly/3nNMpfH
as well as a video overview describing the platform (timepoint 4:50): https://bit.ly/3r5GrZr

As far as the time to complete the loop, this is hard to quantify because it is highly dependent on the complexity of the structure being segmented and the amount of training data. While the annotation tools available in HistomicsUI are very robust, we feel that the ability to quickly correct computationally predicted boundaries is better implemented in Aperio ImageScope, which is why we have included tools for round trip ingestion and export between HistomicsUI and Imagescope, these are described in the METHODS Functionality section. During the process of developing the glomerular model, we performed annotation correction using Aperio ImageScope.

However, in the future, we aim to develop tools which aid and speed up the annotation of WSIs within the HistomicsUI interface. We describe some of these future directions in the DISCUSSION section in the third paragraph.

5. Line 208-213. "…the resulting computationally estimated scores were correlated with the manual visual estimation." and Figure 4c and Supp. Figure 4. Pearson correlation coefficient is not a valid statistical method for this comparison. Pearson correlation coefficient only examine the correlation, but here the aim is the reproducibility of percent IFTA. Please consider intraclass correlation coefficient instead. If ICC is used, please do specify the chosen "Mode", "Type", and "Definition". Also, please provide raw data for the statistics in supplementals.

*Thank you for pointing this out to us, you are correct! We have updated **Fig. 4** and **Supp. Fig. 4** with ICC values computed using two-way random effects, absolute agreement, and single rater/measurement. This is reflected in the subsection Statistical analysis under the section METHODS and also as part of the caption of **Supp. Fig. 4.***

*We have also provided a table with the raw percent IFTA data from the pathologists and computational models in **Supp. Table 3**.*

6. Line 263. It would be better if the authors mention the size of training data for glomerulus segmentation model in the first paragraph of Glomerular segmentation – scalability in RESULTS to emphasize it's a scalability test.

We have added a reference to the number of pixels (1.8 trillion) the training set contained in this paragraph.

7. Line 280. "Predictably, performance on the independent test datasets GlomTestSet 2 & 3 was lower than the holdout dataset, albeit visual manual assessment of glomerular segmentations appeared to be consistent with expert opinion." Please clarify this sentence.

We have changed the wording of this sentence, and emphasized that:

1) *the independent test set slides originated from institutions separate from the training data.*
2) *Visual assessment of the independent test set segmentations by expert pathologists was favorable. Namely, eight renal pathologists in our authorship team anonymously agreed that the segmented glomerular boundaries match their definition of glomerulus for pathologic assessment.*

8. Line 284. Here in DISCUSSION and in VESSLE SEGMENTATION – adaptability in RESULTS (and in Figure 3a), both Cohen's kappa and MCC were used, which is quite confusing. Cohen's kappa was used in DISCUSSION and in the figure legend of Figure 3a (line 737-743), but the y-axis in Figure 3a showed MCC. Please consider using homogenous expression. Either Cohen's kappa or MCC showed very similar values between models constructed from Glomerulus-pretrained model or ImageNet-pretrained model. Therefore, "When testing the effectiveness of transfer learning, we found that adapting the glomerulus model for segmenting glomeruli, arteries, and arterioles using the VesselTrainSet, did not perform as well as when the same dataset was trained using the ImageNet model as the starting point (albeit very marginally)." might be an overstatement, unless the authors could show that the results from two pretrained models are statistically different. The following discussion in the paragraph was based on the assumption that the ImageNet-pretrained model is truly better. It is advised that the authors re-write this paragraph.

Thank you for pointing out our lack of consistency regarding MCC and kappa. Originally, we had chosen to include the kappa score as it is a summary metric (one number for a multi class segmentation problem). However, we agree that this is confusing to the reader, and have revised these sections to include only MCC scores (with one MCC score for each class – glomeruli, arterioles, & arteries).

We also acknowledge that our claim of the ImageNet model being better than the Glomeruli model was an overstatement. We have revised and toned down the sections that refer to this comparison, stating that the two models are comparable. We still feel that our choice to make the ImageNet model the default model for performing transfer learning is valid. This model performs as well as the Glomeruli model and we feel that due to the diversity of the ImageNet dataset, this model may offer a better feature generalizability when applied to non-renal tissues. In future work this is something that we think would be very interesting to explore in greater detail!

9. Line 401. Did the KPMP WSI dataset for testing of ITFA model follow the selection criteria mentioned in Line 385-389?

Yes, it did. We have clarified this in the text.

10. Line 530. As mentioned, Pearson correlation coefficient is not a valid statistical method for the comparison.

We have addressed this comment and now use ICC – see the response above.

11. Line 713. Figure 2b. Please use Tissue pixels for x-axis instead. It is more relevant to consider the size of “tissue” pixels rather than “whole image” pixels.

The reviewer makes a valid point, however respectfully, we believe that the figure better serves the paper as is. The color coding of the points depicts the number of tissue pixels in the slide (we have updated the figure legend to reflect that these are depicted in a log10 scale).

The speed of Histo-cloud’s segmentation of WSIs is largely derived from the on-the-fly processing of regions of interest from WSIs, therefore during prediction, patches from the WSI need to be processed streamed to the GPU for processing, before being stitched back together into the final WSI sized prediction. Part of the intention of this figure is to depict the programmatical overhead when opening and streaming data from very large WSIs – which is slower than the case for smaller slides (in terms of total slide pixels / dimension).

We chose to use tissue pixels as color and total slide pixels as the x axis, as we believe this is the best way to show this complex relationship. When the data is represented in this way you can see that the slide size has an effect on the segmentation time in addition to the tissue size (even when the tissue area is held constant). This can be seen by the positive slope of the color gradient of the points. This relationship is discussed in the RESULTS section under GLOMERULAR SEGMENTATION – scalability in the third paragraph.

Reviewer #2 (Remarks to the Author):

The authors propose Histo-Cloud: a tool for segmentation of WSIs with an interactive graphical user interface. Histo-Cloud builds functionality on top of the HistomicsTK interface by enabling CNNs to segment regions within WSIs. The tool also enables features to be extracted from segmented regions for further analysis. The paper is well organised and is easy to follow and understand throughout.

We thank Reviewer 2 for their kind description, and have addressed the points raised below individually. These revisions in the text are highlighted in Green.

Overall, the method is interesting, and it aims to reduce the effort needed for users to train segmentation-

based models. The entry level required for developing deep learning models is lowered by packaging the software in the form of a GUI. This is good, but I would like to hear more on what controls are in place to ensure models are trained correctly. For example, pathologists may not annotate exhaustively, which is typically required for supervised segmentation. What happens in this case? I would also like to know more on what the GUI displays during model training. Are any statistics or plots shown? Also, can you add more information on how a user would connect the application to their own compute?

Thank you for your interest in our method!

To touch on the points that you brought up, our method allows the user to set the hyperparameters used for training, if the user would like we have a control that allows sections of the WSI to be excluded from the training process. While we originally envisioned this ability to be useful for the exclusion of ambiguous regions from the training set, it could also be used to exclude a large portion of a WSI where the end user only wants to do partial annotation. See more details at https://bit.ly/3r5GrZr discussing a video overview of Histo-Cloud, and at https://bit.ly/3nNMpfH discussing a documentation for using this tool. Further, we added additional discussion under the section METHODS – Functionality, as well as ***Supp Fig. 5.***

As far as monitoring the progress, the user is able to view the command line output from the running job if they choose, which includes useful information about the training progress. This is discussed in the video overview [https://bit.ly/3r5GrZr.](https://bit.ly/3r5GrZr)

For example, the snap below shows the backend command line progress during training:

```
I0110 05:55:41.244129 140571384850240 learning_save_zip.py:449] global step 10: loss = 1.4587 (0.178 sec/step)
INFO:tensorflow:global step 20: loss = 1.4349 (0.178 sec/step)
I0110 05:55:43.458451 140571384850240 learning_save_zip.py:449] global step 20: loss = 1.4349 (0.178 sec/step)
INFO:tensorflow:global step 30: loss = 1.4019 (0.178 sec/step)
I0110 05:55:45.441932 140571384850240 learning_save_zip.py:449] global step 30: loss = 1.4019 (0.178 sec/step)
INFO:tensorflow:global step 40: loss = 1.3712 (0.172 sec/step)
I0110 05:55:47.433856 140571384850240 learning_save_zip.py:449] global step 40: loss = 1.3712 (0.172 sec/step)
INFO:tensorflow:global step 50: loss = 1.3334 (0.176 sec/step)
I0110 05:55:49.336293 140571384850240 learning_save_zip.py:449] global step 50: loss = 1.3334 (0.176 sec/step)
INFO:tensorflow:global step 60: loss = 1.3183 (0.171 sec/step)
I0110 05:55:51.389750 140571384850240 learning_save_zip.py:449] global step 60: loss = 1.3183 (0.171 sec/step)
INFO:tensorflow:global step 70: loss = 1.2716 (0.171 sec/step)
I0110 05:55:53.218645 140571384850240 learning_save_zip.py:449] global step 70: loss = 1.2716 (0.171 sec/step)
INFO:tensorflow:global step 80: loss = 1.2318 (0.177 sec/step)
I0110 05:55:55.216961 140571384850240 learning_save_zip.py:449] global step 80: loss = 1.2318 (0.177 sec/step)
INFO:tensorflow:global step 90: loss = 1.1999 (0.174 sec/step)
I0110 05:55:57.316627 140571384850240 learning_save_zip.py:449] global step 90: loss = 1.1999 (0.174 sec/step)
INFO:tensorflow:global step 100: loss = 1.1499 (0.179 sec/step)
I0110 05:55:59.513291 140571384850240 learning_save_zip.py:449] global step 100: loss = 1.1499 (0.179 sec/step)
INFO:tensorflow:global step 110: loss = 1.1009 (0.178 sec/step)
I0110 05:56:01.340224 140571384850240 learning_save_zip.py:449] global step 110: loss = 1.1009 (0.178 sec/step)
INFO:tensorflow:global step 120: loss = 1.1124 (0.176 sec/step)
I0110 05:56:03.309723 140571384850240 learning_save_zip.py:449] global step 120: loss = 1.1124 (0.176 sec/step)
INFO:tensorflow:global step 130: loss = 0.9426 (0.187 sec/step)
I0110 05:56:05.442852 140571384850240 learning_save_zip.py:449] global step 130: loss = 0.9[illegible]
INFO:tensorflow:global step 140: loss = 0.9053 (0.181 sec/step)
```

2.6% Train Segmentation Network on...

Train Network: Loss: 0.9425593018531799

Further the snap below shows the command line output at the backend showing progress during prediction:

```
2022-01-10 05:32:12.277183: I tensorflow/core/common_runtime/gpu/gpu_device.cc:1325] Created TensorFlow device (/job:localhost/replica:0/task:0/dev
2022-01-10 05:32:14.155375: I tensorflow/stream_executor/platform/default/dso_loader.cc:44] Successfully opened dynamic library libcudnn.so.7
2022-01-10 05:32:16.034736: I tensorflow/stream_executor/platform/default/dso_loader.cc:44] Successfully opened dynamic library libcublas.so.10.0
Working on [K1 PAS.svs] patch: [1 of 107]
Working on [K1 PAS.svs] patch: [2 of 107]
Working on [K1 PAS.svs] patch: [3 of 107]
Working on [K1 PAS.svs] patch: [4 of 107]
Working on [K1 PAS.svs] patch: [5 of 107]
Working on [K1 PAS.svs] patch: [6 of 107]
Working on [K1 PAS.svs] patch: [7 of 107]
Working on [K1 PAS.svs] patch: [8 of 107]
Working on [K1 PAS.svs] patch: [9 of 107]
Working on [K1 PAS.svs] patch: [10 of 107]
Working on [K1 PAS.svs] patch: [11 of 107]
Working on [K1 PAS.svs] patch: [12 of 107]
Working on [K1 PAS.svs] patch: [13 of 107]
Working on [K1 PAS.svs] patch: [14 of 107]
```

Keyword arguments:

```
{
  "container_args": [
    "SegmentWSI",
    "--batch_size",
    "1",
    "--gpu",
    "0",
    [illegible]
```

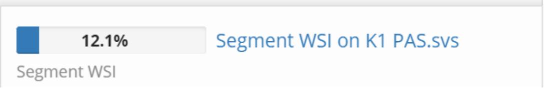

However, we recognize that for less experienced users this above may seem daunting. We have therefore added a progress bar to the prediction and training plugins which pops up in the lower right corner of the screen at the user side. During training, this progress bar also displays the network loss at regular intervals. These progress bars are also visible at the backend displaying progress as demonstrated in the snaps above. In the future we would love to incorporate more advanced graphical features into the GUI, something like an integration with Tensorboard to display the training loss as a curve would be very useful.

*We have added **Supp Fig 5** to highlight these abilities (region exclusion and progress monitoring). See also discussion under the section METHODS – Functionality*

As far as how a user would add this application to their own compute: This application is built on the Digital Slide Archive (DSA), an open source tool from Kitware Inc. It can be deployed on any system capable of running the DSA, Athena – our deployed instance is running in an OpenStack instance rented through SUNY Buffalo, however, local deployment on Linux systems is possible (we have tested this on Ubuntu 18.04 and 16.04), as well as deployment using AWS. We have included installation instructions in the github page for Histo-Cloud. Note that we distribute Histo-Cloud as a docker container image. Detailed installation instructions for the DSA are available from Kitware, and pulling plugins like Histo-Cloud via DSA is discussed under DSA support page available via Kitware. See the relevant links under the section Results – HISTO-CLOUD. As part of our upcoming grant, we are planning to start a Slack channel for users to address their inquiries including queries on installation.

Is there an option to export the trained model? After all, a user may want to use the model in a downstream application, but not necessarily in Histo-Cloud. Also, it would be good if the user could add their own custom model rather than relying on the DeepLab v3 architecture. For example, a user may have their own developed model that performs well, but they want to improve the performance using Histo-Cloud. Why was TensorFlow selected? If using the application for improving model performance, then users may want to also explore PyTorch models – please give justification for the choice of deep learning library.

***Exporting models:** Histo-cloud manages saved models by zipping the files containing the trained model parameters (ckpt files) into a single zip file. This zip file is accessible by the user and can be used for prediction internally in Histo-Cloud or downloaded and unzipped for use in another downstream application. An example of a saved model is available here:* [*https://athena.ccr.buffalo.edu/#collection/5fa17ef9e8737fef305946fe/folder/5fa17f11e8737fef305946ff*](https://athena.ccr.buffalo.edu/#collection/5fa17ef9e8737fef305946fe/folder/5fa17f11e8737fef305946ff)

Architecture choices: *We acknowledge the appeal of a generalized system for users to deploy models for deep learning on WSIs. HistomicsUI (the open source tool available from Kitware Inc.) allows a plugin to be created from dockerized python code. This is the tool that we used to create our plugins. DSA support page available via Kitware has directions discussing how to import new plugins. We discuss this further at the end of this response.*

At the beginning of this project we set out to make an accessible segmentation tool for the digital pathology community. Towards this goal our engineering effort was focused on creating a reliable and scalable tool that is capable of efficient WSI segmentation. We chose the DeepLab architecture because of its state-of-the-art segmentation performance, and because we had worked with it in the past.

The selection of the particular deep learning architecture (TensorFlow) was based on the availability of source code for the DeepLab V3+ architecture. The official implementation of this code supports multi GPU training and pre-trained models (such as the ImageNet model used in this paper for transfer learning) are available from the Google Model Zoo. While the Athena server only has a single GPU, we have tested Histo-cloud on more powerful servers using multiple GPUs during training.

The source code for Histo-cloud includes our modified DeepLab codebase. This is capable of streaming data directly from WSIs for training or prediction and has been carefully tuned to be fast, memory efficient, and handle unique challenges of segmenting WSIs, including large class imbalances. A detailed breakdown of the logic used for on-the-fly region of interest selection during training is highlighted in Supplemental figure 1. Using this code advanced users can run training and prediction from a command line interface if desired.

While the engineering decisions made to create and optimize Histo-cloud require the use of TensorFlow and DeepLab, the development of a plugin similar to Histo-cloud which uses another deep learning library such as PyTorch is possible. We believe that our source code will serve as a good example of how to adapt and package a Deep learning tool as a plugin using HistomicsUI regardless of the model or architecture used. The developer needs to ensure that their segmentation tool that uses PyTorch works via command line interface in the server that hosts DSA. One simple way to import this tool is pushing first a dockerized image of the tool in Docker Hub, and then the image can be pulled using DSA using the direction provided in DSA support page of Kitware.

I would like more emphasis to be placed on the platform rather than the segmentation models. I understand that specific applications should be highlighted, but state with more clarity why Histo-Cloud enabled this.

We agree that the HistomicsUI platform is extremely interesting and useful to the digital pathology community. Our Histo-Cloud tool extends the functionality of HistomicsUI enabling easy development and usage of segmentation models on WSIs. Describing a tool like this in a journal article is challenging and as the reviewer mentioned specific applications should be highlighted.

Our goal in this paper was to highlight the applications of Histo-cloud using use cases from renal pathology (the specialty we are familiar with). Throughout this paper we have crafted experiments which demonstrates the performance characteristics of Histo-Cloud:

- *Our Glomerular segmentation example shows the scalability of the tool when training and testing models on very large WSI datasets. The highly optimized on-the-fly processing of WSI patches for training and testing (unique to Histo-Cloud) is responsible for this scalability. This is described in detail in Supp Fig 1.*
- *Our Vessel segmentation example shows the ability of Histo-Cloud to perform multi class segmentation as well as showing the benefits of transfer learning. As part of this study we trained a model using 15989 WSIs from the GTEx dataset, again made possible by the on-the-fly processing of WSI patches during training.*
- *Our IFTA segmentation example demonstrates the feasibility of using Histo-Cloud for collaborative annotation and model development efforts across institutions. IFTA is a notoriously difficult structure to annotate, as the boundaries are not well defined and there is a great degree of disagreement among pathologists. We believe the ability to easily collaborate in a cloud-based viewer contributed to the success of our IFTA model (which was*

trained using 3 annotators from 3 institutions). The pathologist annotators were able to view each other's annotations and find adjust their annotations for better concurrence on IFTA definition.

- *Finally, the mouse model study shows a potential downstream application of segmentation performed using Histo-Cloud. We believe that Histo-cloud is the ideal tool for such applications because it removes the technical barrier of entry for non-machine learning experts to segment WSIs. This process enables a user with limited computational experience to parse WSIs into biologically relevant compartments for further analysis. We have included a plugin which extracts basic morphological features from segmented structures and is capable of outputting these measurements as a spreadsheet for further analysis.*

We have opened an instance of this tool to the community for exploration / experimentation and feel that this hands-on experience is the best way to demonstrate the abilities of the platform: https://athena.ccr.buffalo.edu/

We point the reviewer to the included documentation of Histo-cloud: https://bit.ly/3nNMpfH
as well as a video overview describing the platform: https://bit.ly/3r5GrZr

How will Histo-cloud be extended? Why is it limited to just segmentation? Hypothetically this should not need to be limited to just segmentation.

Segmentation has been essential for many of the works published by the Sarder Lab, and as a result it seemed like a natural first step for the development of a more accessible tool for computational pathology. Histo-Cloud was the Sarder Lab's first foray into the development of cloud-based tools for computational pathology and during its development we aimed to keep the tool as generalizable and scalable as possible for the widest possible applicability. However, this generalizability and the wide gamut of tests with diverse datasets slowed down the time to publication for Histo-cloud.

Using the codebase of Histo-cloud as a template we have developed several other works around the same core philosophy. PodoSighter – a more targeted cloud-based tool for identifying podocyte nuclei from renal brightfield WSIs was recently published in the Journal of the American Society of Nephrology: https://jasn.asnjournals.org/content/32/11/2795.abstract. PodoSighter is also freely available in Athena, the server that was setup to host our Histo-cloud demo. Glomerular Segmentation is required prior to PodoSighter analysis, which is accomplished using Histo-Cloud.

In the future we would also like to extend Histo-cloud to include instance segmentation and develop a plugin for classification of segmented regions of interest. We have added a sentence at the end of the discussion section stating this intention.

We are also preparing a manuscript on federated segmentation of WSIs, where we use Histo-Cloud and the REST API of HistomicsUI to perform federated training of segmentation models across physically distinct servers. A pre-print of this manuscript is available here: https://www.biorxiv.org/content/10.1101/2021.08.17.456679v1.

Are there different options for visualisation? Such as the colour of overlay and transparency? Does the segmentation work on the entire WSI or on image regions within the WSI?

Yes, the user can choose to adjust options for visualization, including the overlay color and transparency. This ability is documented in our guide available here: https://bit.ly/3nNMpfH (step 14 - on page 5).

Histo-Cloud segments the entire WSI and outputs computational annotations that are viewable directly on the slide (by creating annotation layers). Since WSIs are too large to fit into memory, training and prediction are accomplished by considering regions of interest from the WSI. For training these regions are selected, extracted, and streamed directly from the WSIs which we have found greatly enhances the efficiency. During prediction, patches from the WSI are once again extracted on the fly but from a gridded pattern so that all the regions containing tissue are analyzed, the resulting predictions are stitched back together to give a prediction on the entire WSI. This implementation is described in the METHODS – Training & Testing section.

We point the reviewer to the included documentation of Histo-cloud: https://bit.ly/3nNMpfH
as well as a video overview describing the platform: https://bit.ly/3r5GrZr

An example of a computationally segmented slide can be viewed here:

https://athena.ccr.buffalo.edu/histomics#?image=606f7a9ccf76438149385d06&bounds=132%2C-941%2C30902%2C30437%2C0

The annotation layers can be viewed by expanding the "other" section under the Annotations tab on the right.

Reviewers' comments:

Reviewer #1 (Remarks to the Author):

I think the manuscript is now almost ready for publication. There is a minor point. In Supplementary Table 3 it mentions "For the Intraclass correlation coefficient calculation in Fig. 4 and Supp Fig. 4 the percent IFTA scores obtained by the computational models were also rounded to the nearest 10%." I don't see the reason for this rounding procedure. There is no need to make the model's interpretation like pathologists' interpretation, i.e., providing a percent IFTA score to the nearest 10 percent. However, I find that all ICCs between Pathologists and the Combined full model slightly increase after this rounding procedure. It would be interesting to investigate if this is a true phenomenon or just coincidence. Nonetheless, it won't alter the overall claim of this paper.

Reviewer #2 (Remarks to the Author):

I thank for authors for taking time to address my main concerns/questions regarding the submission. I think that the revision has led to an improvement of the manuscript.

I have a few more points that I would like to be addressed:

In the abstract, it is mentioned that scientists 'often face prohibitive challenges in using these tools to their full potential'. I think this is a bit ambiguous - can you reword to explain why they can't use the tools to their full potential, which leads more smoothly on to the next sentence.

Some of the figures look a bit cluttered. For example, in Fig 2 I would prefer the segmentation results to all be below the plots and be better aligned. Also consider using a thicker line to highlight the segmentation results, as they are quite hard to see. For Fig 3, it is hard to know which images belong to (b) and which ones belong to (c). These need to be organised more clearly.

I would like further clarification on the interactive learning strategy. For example, does the model completely rely on images annotated in the viewer? Or can you incorporate additional external data? This may be beneficial if users want to leverage already existing datasets and use Histo-Cloud for refinement purposes.

Reviewers' comments:

We again thank the reviewers for their constructive comments, we have addressed these pointwise below:

Reviewer #1 (Remarks to the Author):

I think the manuscript is now almost ready for publication. There is a minor point. In Supplementary Table 3 it mentions "For the Intraclass correlation coefficient calculation in Fig. 4 and Supp Fig. 4 the percent IFTA scores obtained by the computational models were also rounded to the nearest 10%." I don't see the reason for this rounding procedure. There is no need to make the model's interpretation like pathologists' interpretation, i.e., providing a percent IFTA score to the nearest 10 percent. However, I find that all ICCs between Pathologists and the Combined full model slightly increase after this rounding procedure. It would be interesting to investigate if this is a true phenomenon or just coincidence. Nonetheless, it won't alter the overall claim of this paper.

We thank the Reviewer for this comment. During the preparation of these figures we debated if the computational percent IFTA scores should be rounded, ultimately deciding to round the scores. However, upon the reviewer's suggestion we have recalculated the ICCs without rounding the computational predictions and updated the confusion matrices in both figures.

Towards the reviewers point about the increase in the ICCs between the Pathologists and Combined Full model, we believe that this is simply an artifact of the rounding procedure. However intuitively this makes sense, as the network (if performing properly) will make percent IFTA predictions that are close to the pathologist derived values. If the average error is less than 5% rounding to the nearest 10% will increase the agreement and therefore the ICC metric between the computational model and the Pathologists. We believe that further investigation into this phenomenon is beyond the scope of this paper, but it would be very interesting to see if the trend is continued for a larger sample size.

Reviewer #2 (Remarks to the Author):

I thank for authors for taking time to address my main concerns/questions regarding the submission. I think that the revision has led to an improvement of the manuscript.

I have a few more points that I would like to be addressed:

In the abstract, it is mentioned that scientists 'often face prohibitive challenges in using these tools to their full potential'. I think this is a bit ambiguous - can you reword to explain why they can't use the tools to their full potential, which leads more smoothly on to the next sentence.

We have reworded this sentence to clarify that Pathologists and Biological Scientists often do not have computer programming experience, and struggle with setup, and usage of machine learning tools which rely on command line interfaces.

Some of the figures look a bit cluttered. For example, in Fig 2 I would prefer the segmentation results to all be below the plots and be better aligned. Also consider using a thicker line to highlight the segmentation results, as they are quite hard to see. For Fig 3, it is hard to know which images belong to (b) and which ones belong to (c). These need to be organised more clearly.

We thank the reviewer for their comments about figure clarity. Looking with fresh eyes we see the issues.

We have updated Fig 2 with thicker lines. The images in Fig 2 were kept at a consistent resolution, we tried to add a wide variety of glomerular morphology to the figure and felt the best way to do this was to crop the images tightly to the glomeruli. The misalignment is an artifact of this intention, and respectfully we do not feel that it should be changed.

We have also placed colored outlines around the images in Fig 3 to better separate (b) and (c).

I would like further clarification on the interactive learning strategy. For example, does the model completely rely on images annotated in the viewer? Or can you incorporate additional external data? This may be beneficial if users want to leverage already existing datasets and use Histo-Cloud for refinement purposes.

We have added a subsection to ***Methods*** titled: ***Iterative Learning & Annotation Ingestion***. Here we briefly clarify the iterative learning strategy. We also address how a user could incorporate additional external data which has been previously annotated. Specifically, we highlight the *<IngestAperioXML>* plugin. If an advanced user needs to import annotations in another format we also link several scripts included in the source code which can help with annotation conversion import and management.

REVIEWERS' COMMENTS:

Reviewer #1 (Remarks to the Author):

The authors have addressed all the concerns. I appreciate their work and think the manuscript is now ready for publication.

Reviewer #2 (Remarks to the Author):

I am satisfied that the reviewers have addressed my comments.
